# The MYBL2-GTSE1 axis promotes laryngeal squamous cell carcinoma progression by regulating PI3K/AKT-dependent glycolytic reprogramming

**DOI:** 10.1080/15384047.2026.2648193

**Published:** 2026-03-22

**Authors:** Yiyin Liang, Cansi Wang, Tianjiao Ren, Bo Zhang, Yaqi Liu, Rong Fu, Juan Feng

**Affiliations:** a Department of Otorhinolaryngology, The First Affiliated Hospital of Xinjiang Medical University, Urumqi, Xinjiang, People's Republic of China

**Keywords:** Laryngeal squamous cell carcinoma, MYBL2, GTSE1, PI3K/AKT signaling pathway, glycolysis, metabolic reprogramming

## Abstract

**Background:**

Laryngeal squamous cell carcinoma (LSCC) is a common head and neck malignancy with poor prognosis. The role of MYBL2, an oncogenic transcription factor, in the glycolytic reprogramming of LSCC remains unclear.

**Methods:**

We integrated RNA-sequencing with public databases (TCGA, GEO) and tissue microarrays to assess MYBL2 expression and its clinical significance. Transcriptional regulation was verified by ChIP-qPCR and luciferase reporter assays. Signaling pathways and metabolic profiles were examined using Western blotting and Seahorse analysis (ECAR/OCR). Biological functions were evaluated by *in vitro* functional assays and *in vivo* xenograft models in female BALB/c nude mice.

**Results:**

MYBL2 was significantly overexpressed in LSCC tissues and correlated with poor prognosis. Mechanistically, MYBL2 directly activates GTSE1 transcription. This regulation stimulates PI3K/AKT signaling to upregulate key glycolytic proteins (PKM2, HK2, GLUT1, LDHA), thereby driving metabolic reprogramming characterized by elevated glycolysis (ECAR) and suppressed mitochondrial respiration (OCR). Functionally, MYBL2 overexpression enhanced the proliferation, migration, and invasion of LSCC cells *in vitro* and promoted tumor growth *in vivo*. Importantly, these oncogenic effects were effectively reversed by GTSE1 knockdown or PI3K inhibition with LY294002, validating the pathway's functional significance.

**Conclusion:**

The MYBL2-GTSE1 axis promotes LSCC progression through PI3K/AKT-mediated metabolic reprogramming, representing a promising therapeutic target.

## Graphical abstract

**Figure:**
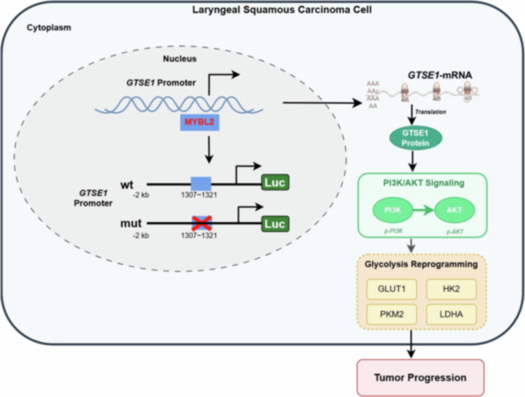

Schematic illustration of the MYBL2/GTSE1/PI3K-AKT axis promoting glycolytic reprogramming and LSCC progression.

## 1. Introduction

Laryngeal cancer represents one of the most common malignancies in the head and neck region^1^ and is the second most frequent respiratory system tumor after lung cancer, with squamous cell carcinoma accounting for 95%−98% of cases.^2^^,^^3^ According to the Global Burden of Disease 2021 data, approximately 200,000 new laryngeal cancer cases and 117,000 deaths were reported worldwide,^4^ posing a significant public health challenge.^5-7^ Despite advances in laryngeal squamous cell carcinoma (LSCC) diagnosis and treatment, patient outcomes remain suboptimal.^8^ Particularly for advanced-stage patients (clinical stages III and IV), the 5-year overall survival rate is approximately 50%, even with multidisciplinary approaches including surgery, radiotherapy, and chemotherapy.^9-11^ Moreover, immune checkpoint inhibitors demonstrate modest objective response rates of 15−20%,^12^ underscoring the need for additional therapeutic strategies. Therefore, a deeper understanding of the molecular mechanisms underlying LSCC development and progression is important for identifying therapeutic targets.

The Warburg effect, characterized by enhanced aerobic glycolysis, drives LSCC pathogenesis through metabolic reprogramming.^13^^,^^14^ This metabolic shift produces both rapid ATP generation and key intermediates essential for tumor proliferation and metastasis.^13^^,^^15^^,^^16^ Such metabolic reprogramming is driven by specific transcriptional regulators. MYB Proto-Oncogene Like 2 (MYBL2), an essential member of the MYB family of transcription factors, plays important roles in cell cycle regulation,^17^ DNA damage repair,^18^^,^^19^ and inhibition of apoptosis.^20^ Our analysis of public data from The Cancer Genome Atlas (TCGA) and our own sequencing data revealed that MYBL2 is upregulated in LSCC and is significantly correlated with poor prognosis. In this context, we investigated the role of MYBL2 in LSCC metabolism. Recent studies have begun to link MYBL2 to metabolic regulation; for instance, the long non-coding RNA MALAT1 has been shown to remodel the glucose metabolic profile of tumor cells by activating the MYBL2-mTOR signaling axis.^21^ Additionally, MYBL2 has been shown to bind directly to the OPA3 promoter, promoting glycolytic switching and malignant phenotypes in hepatocellular carcinoma.^22^ Nevertheless, the specific mechanisms by which MYBL2 may participate in glycolysis in LSCC remain largely unknown.

To address this question, we examined the signaling pathways that regulate glycolytic metabolism in LSCC. We focused on the PI3K/AKT pathway, a central regulator of cellular growth, survival, and metabolism that plays an important role in glycolytic reprogramming.^23^^,^^24^ PI3K/AKT signaling promotes the Warburg effect by phosphorylating metabolic transporters and enzymes (GLUT1, HK2, PKM2). Additionally, this pathway modulates key transcriptional regulators such as MYC and HIF-1α.^25^ Given the central role of PI3K/AKT in metabolic reprogramming, we sought to identify potential mediators linking MYBL2 to this pathway. Studies have demonstrated that G2 and S phase expressed protein 1 (GTSE1), a cell cycle regulatory molecule, activates AKT/mTOR signaling and promotes tumor progression.^26^ Moreover, GTSE1 downregulation suppresses the Warburg effect and reduces LDHA expression,^27^ suggesting a role in metabolic regulation. However, whether GTSE1 serves as a molecular bridge connecting MYBL2 to the PI3K/AKT pathway remains unknown.

Our study aims to investigate the biological functions of MYBL2 in LSCC and to elucidate the potential role of GTSE1 in mediating MYBL2-driven metabolic reprogramming. Understanding this mechanism may provide novel therapeutic strategies for LSCC treatment.

## 2. Materials and methods

### 2.1. Tumor specimens

This study analyzed both surgical specimens and commercial tissue microarrays (TMAs). Twenty pairs of LSCC tumors and adjacent normal tissues were collected during routine surgery at the First Affiliated Hospital of Xinjiang Medical University from March 2020 to December 2022. No patients received chemotherapy, radiotherapy, or other related antitumor therapies before surgery. Detailed clinical data are provided in ***Supplementary File 2. Tab. S1.*** Additionally, this study analyzed two commercial tissue microarrays (TMAs): YP-RNS804 (Hefei Leagor Biotech Corp., Hefei, China) and HN049La01 (Zhongke Guanghua Intelligent Biotech Co., Ltd., Xi'an, China). The former comprised 80 tissue cores (60 tumor, 20 adjacent normal), while the latter contained 49 cores (43 tumor, 6 adjacent normal). Detailed specifications of these TMAs are listed in ***Supplementary File 1***. *Tab. S5,* while the corresponding clinicopathological characteristics are listed in ***Supplementary File 2. Tab. S2–3.*** Tumor staging was performed according to the American Joint Committee on Cancer (AJCC) TNM Staging System (8th edition). This study was approved by the Ethics Committee of the First Affiliated Hospital of Xinjiang Medical University and conducted in accordance with the Declaration of Helsinki. Written informed consent was obtained from all participants.

### 2.2. RNA-sequencing data and bioinformatics analysis

The transcriptomic data in this study were derived from two sources: (i) RNA sequencing data from 20 pairs of LSCC and adjacent normal tissues collected at our institution, and (ii) validation datasets from public databases, including the TCGA-LSCC RNA-seq dataset (https://portal.gdc.cancer.gov/) and the GSE59102 microarray dataset (https://www.ncbi.nlm.nih.gov/gds/?term=GSE59102). Raw RNA-seq data were normalized using the edgeR package, while microarray data were preprocessed using the limma package. To identify robust candidate genes, we implemented a multi-stage screening strategy. Differentially expressed genes (DEGs) (***Supplementary Table S1***) were identified using the criteria of |log_2_ fold change| > 1 and *P*-value < 0.05. The candidate list was further refined by intersecting these DEGs with those from the TCGA and GSE59102 datasets. The overlapping candidates were subsequently subjected to univariate Cox regression analysis to evaluate their prognostic significance, with the gene exhibiting the most significant hazard ratio selected for further validation (***Supplementary Table S2***). Gene Set Enrichment Analysis (GSEA) was conducted using the clusterProfiler R package.^28^ Samples were stratified into high and low-expression groups based on the median gene expression. Genes were ranked according to the log_2_ fold change values. The reference gene sets (c2.cp.reactome) were retrieved from the MSigDB database.^29^^,^^30^ The Normalized Enrichment Score (NES) was used to quantify the magnitude of pathway enrichment. Statistical significance was defined as *P*-value < 0.05 and False Discovery Rate (FDR) < 0.25. All data acquisition and usage complied with relevant database policies.

### 2.3. Cell culture, lentiviral transduction, and drug treatment

The Human Primary Laryngeal Mucosal Epithelial Cells (HPLMEC; RPC-050; RUNTOGGN) and four LSCC cell lines—AMC-HN-8 (TCHu262; CAS Cell Bank), TU138 (BTCC-1192; BTCC), TU686 (ORC0873; Chemical Book), and TU212 (SNL-497; SUNCELL)—were used in this study. (Details are listed in ***Supplementary File 1. Tab. S6***). AMC-HN-8 and TU138 were selected for functional studies based on their higher MYBL2 expression levels. AMC-HN-8 was established from a metastatic lymph node of a patient with LSCC,^31^ while TU138 is a well-characterized and extensively validated LSCC cell line that has been widely used in laryngeal cancer research.^32^ Both cell lines were routinely tested for mycoplasma contamination and confirmed to be mycoplasma-free. (Details are provided in ***Supplementary File 3***.) Lentiviral vectors for MYBL2 and GTSE1 knockdown, as well as MYBL2 overexpression, were obtained from Shanghai GeneChem Co., Ltd. Cells were seeded in 12-well plates (5 × 10^4^ cells per well) and transduced with lentivirus at a multiplicity of infection (MOI) of 30. At 72 h post-transduction, cells were selected with puromycin (ST551; Beyotime) for 14 days. Lentiviral transduction efficiency was assessed by fluorescence microscopy (***Supplementary File 1. Fig. S1 and Fig. S2)***. The functional efficacy of shGTSE1 was further confirmed through rescue experiments (***Supplementary File 1. Fig. S6***). At 70–80% confluence, cells were treated with the PI3K inhibitor LY294002 (10 μM; S1737, Beyotime) or the AKT inhibitor MK-2206 (5 μM; S1078, Selleck) for 24 h. Control groups were treated with an equivalent volume of the vehicle (DMSO; Y026158, Beyotime). Detailed specifications of all reagents and equipment used in these experiments are listed in ***Supplementary File 1. Tab. S7–S8.***

### 2.4. Reverse transcription quantitative PCR (RT-qPCR)

Total RNA was extracted using TRIzol reagent (R0016; Beyotime) and reverse transcribed into cDNA using the RevertAid First Strand cDNA Synthesis Kit (K1621; Thermo Fisher Scientific). qPCR was performed on an ABI 7500 Fast System using Bestar SYBR Green Mastermix (DBI-2073; DBI Bioscience). Relative gene expression levels were calculated using the 2^-ΔΔCT^ method. All primer sequences can be found in ***Supplementary File 1. Tab. S4.***

### 2.5. Western blot (WB)

Total cellular proteins were extracted with RIPA buffer (P0013B; Beyotime) supplemented with a protease and phosphatase inhibitor cocktail (P1045; Beyotime). Proteins were separated by SDS-PAGE and transferred onto PVDF membranes (IPVH00010; Merck). After blocking in 5% milk/TBST (ST828; Beyotime), membranes were incubated with primary antibodies overnight at 4 °C, followed by incubation with secondary antibodies. Protein bands were visualized using ECL reagents (P0018S; Beyotime). All antibodies used are listed in ***Supplementary File1. Tab. S1 and Tab. S2.***

### 2.6. Colony formation assay

Cells were plated in 6-well plates (500 cells per well) and allowed to grow for 8 days. Colonies were fixed with 4% paraformaldehyde (B1057; Applygen), stained with 1% Crystal Violet (G1062; Solarbio), and photographed. Colonies containing >50 cells were counted under a microscope.

### 2.7. Wound healing assay

Cells were grown to confluence and treated with 1 μg/mL mitomycin C (50-07-7; MedChem Express) in serum-free medium for 1 h. A scratch was made using a 200 μL pipette tip, followed by PBS washing. Wound closure was monitored by microscopy (100×) at 0 h and 48 h post-scratch. The migration rate was calculated as:

$$\mathrm{Migration}\;\mathrm{Rate}\;(\%)=\left[(\mathrm{Widt}h_{0h}\;-\;\mathrm{Widt}h_{48h})/\mathrm{Widt}h_{0h}\right]\times 100\%$$

### 2.8. Cell counting kit-8 (CCK-8) analysis

To assess cell proliferation, 5,000 cells were seeded per well in 96-well plates. Cell viability was monitored daily for 5 days. Briefly, 10 μL of CCK-8 reagent (C0038; Beyotime) was added to each well and incubated for 2 h at 37 °C in the dark. Absorbance readings at 450 nm were taken directly, and these values were used to generate growth curves.

### 2.9. Transwell

Cell invasion was evaluated using 8.0μm Transwell chambers pre-coated with Matrigel (C0383, Beyotime; 1:8 dilution). Cells (5 × 10⁴) in serum-free medium were seeded in the upper chamber, with 20% FBS-containing medium in the lower chamber as a chemoattractant. After 24 h, invaded cells on the lower membrane were fixed with 4% paraformaldehyde, stained with 1% crystal violet, and counted in five random fields (200×).

### 2.10. Flow cytometry assay/apoptosis detection

Apoptosis was analyzed using an Annexin V-APC/PI Apoptosis Kit (E-CK-A217; Elabscience) according to the manufacturer's protocol. Cells (1 × 10⁵) were first incubated with 5 μL Annexin V-FITC for 10 minutes, followed by 5 μL PI for 5 minutes, both at room temperature in the dark. Samples were then diluted to 500 μL with binding buffer for flow cytometric analysis. For positive control validation (***Supplementary File 1. Fig. S4***), cells were pretreated with Staurosporine (1 μM; S1421, Selleck) for 24 h.

### 2.11. Measurement of ECAR and OCR

A Seahorse XF96 Analyzer (Agilent Technologies) was used to assess cellular bioenergetics by measuring extracellular acidification rate (ECAR) and oxygen consumption rate (OCR). ECAR was measured following sequential injections of glucose (10 mM), oligomycin A (1.5 μM), and 2-DG (50 mM). OCR was determined through successive additions of oligomycin A (1.5 μM), FCCP (1.0 μM), and rotenone/antimycin A (0.5 μM). Results were normalized to cell numbers (pmol/min/10⁴ cells). All procedures were performed according to the manufacturer's protocols.

### 2.12. Hematoxylin-eosin (H&E) and immunohistochemistry (IHC)

Tissue morphology was assessed using H&E staining, which involved standard dewaxing and rehydration, followed by sequential staining with hematoxylin (ST2067-20g; Beyotime) and eosin (C0109; Beyotime). IHC staining included dewaxing and rehydration, antigen retrieval, incubation with primary and secondary antibodies, DAB chromogen development (ab64238; Abcam), and hematoxylin counterstaining. Stained slides were digitally scanned and analyzed using CaseViewer software. Two pathologists independently evaluated MYBL2 expression using the H-score method. Protein expression in the tumor microenvironment was quantified by mean optical density (MOD).

### 2.13. Chromatin immunoprecipitation (ChIP) assay

Cells were cross-linked with 1% formaldehyde, followed by glycine quenching. After cell lysis, chromatin was sheared into fragments of 200–500 bp by sonication. The chromatin fragments were then incubated with anti-MYBL2 antibody (18896-1-AP; Proteintech) or rabbit control IgG (AC005; Abclonal) as a negative control, and the immune complexes were captured using protein A-agarose beads (1614813; BIO-RAD). Following elution, reverse cross-linking, and DNA purification, qPCR was performed to quantify the enrichment of the GTSE1 promoter region, with primer sequences listed in ***Supplementary File 1. Tab. S3 and Tab. S4.***

### 2.14. Dual-luciferase reporter assays

To assess the promoter activity of GTSE1, AMC-HN-8 and TU138 cells were co-transfected with a pGL3 firefly luciferase reporter construct containing the GTSE1 promoter (1307–1321 bp) and either a MYBL2 overexpression plasmid or an empty vector, together with the pRL-TK Renilla luciferase vector as an internal control. Luciferase activity was assessed 48 h after transfection using the Dual-Luciferase Reporter Assay Kit (RG027; Beyotime) on harvested cells. The ratio of firefly (pGL3) to Renilla (pRL-TK) luminescence was used to determine relative luciferase activity. All experiments were performed in triplicate.

### 2.15. Animals study

BALB/c-nude mice, which lack mature T lymphocytes and thus permit stable engraftment of human tumor cells, were used as the xenograft model for evaluating *in vivo* LSCC progression. Female mice (4–5 weeks old, 18–22 g) were purchased from Hubei Bairen Biotechnology Co., Ltd. (Wuhan, China; License No. SCXK(E)2021-0027) and maintained under specific pathogen-free (SPF) conditions with free access to food and water. Mice were allowed to acclimatize for one week before tumor cell inoculation, during which body weight and general health status were monitored daily. Mice exhibiting signs of illness or abnormal behavior during this period were excluded prior to group allocation. All animal procedures were approved by the Institutional Animal Care and Use Committee (IACUC) of Xinjiang Medical University (Approval No. A240522-12) and performed in accordance with the National Institutes of Health Guide for the Care and Use of Laboratory Animals.

For xenograft establishment, four stable TU138 cell lines were used: NC+shCtrl, MYBL2+shCtrl, NC+shGTSE1, and MYBL2+shGTSE1. Logarithmic growth phase cells were harvested, resuspended in serum-free 1640(L1039-500; BDBIO), and mixed 1:1 (v/v) with Matrigel Matrix (BD356234; Corning) to enhance tumor engraftment as previously described.^33^^,^^34^ Twelve mice were randomly assigned to four experimental groups (*n* = 3 per group) using a random number generator. Each mouse received a subcutaneous injection of 1 × 10⁶ viable cells in 200 μL into the right flank, with each group receiving the corresponding stable cell line variant, following a protocol established for head and neck cancer xenograft models to ensure reproducible tumor formation.^35^^,^^36^ Due to visible differences in tumor growth, blinding was not feasible during measurements, but data analysis was performed in a blinded manner.

Tumor dimensions were measured every 3–4 d from day 7 post-injection using digital calipers. Tumor volume was calculated as (L × W²)/2, where L and W represent the longest and shortest diameters, respectively. Body weight was recorded simultaneously to monitor overall health. No adverse events were observed during the experimental period. On day 21, to minimize suffering, mice were deeply anesthetized by intraperitoneal injection of 1% pentobarbital sodium (50 mg/kg) until they lost their righting reflex and showed no response to toe pinch. Subsequently, the animals were euthanized by cervical dislocation, and death was confirmed by cessation of heartbeat and respiration. Tumors were then excised, weighed, and photographed. No animals were excluded from the analysis.

### 2.16. Statistical analysis

All statistical analyzes were performed using GraphPad Prism 9.0 and R software 4.2.1. Experimental data from at least three independent replicates are presented as mean ± standard error of the mean (SEM). Data distribution was assumed to be normal, and variances were similar across comparison groups. Pairwise comparisons were conducted using two-tailed t-tests, while multiple group comparisons employed one-way ANOVA with Tukey's Honest Significant Difference (HSD) test. Survival analysis utilized the Kaplan-Meier method with log-rank testing for group comparisons. For TCGA data, differential gene expression was assessed by Wilcoxon rank-sum test and inter-gene correlations by Pearson analysis. Pathway enrichment was determined using GSEA, with significance determined by NES and FDR. Statistical significance was set at *P* < 0.05 (*P < 0.05, **P < 0.01, ***P < 0.001; ns, not significant).

## 3. Result

### 3.1. MYBL2 is upregulated and clinically relevant in LSCC

**Figure 1.**
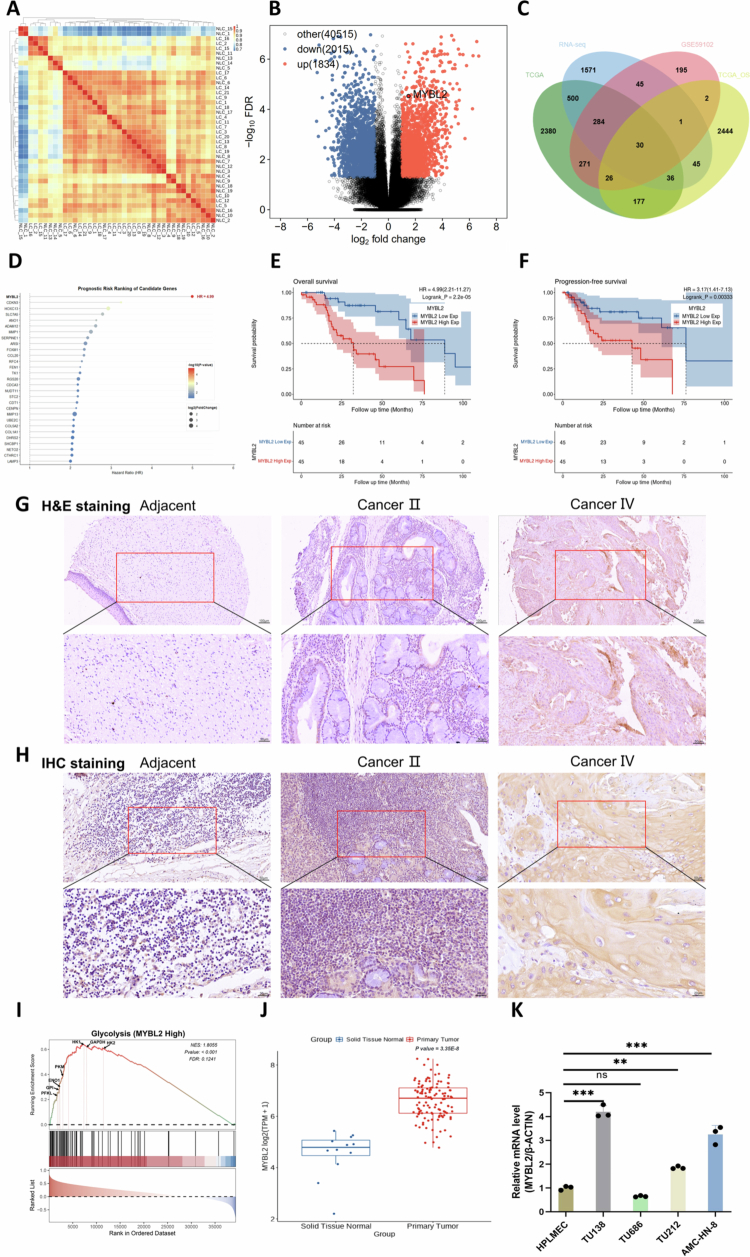
MYBL2 is Upregulated in LSCC and Associates with Poor Prognosis and Regulates Glycolysis. A. Heatmap illustrating the clustering and Pearson correlation analysis of 20 paired LSCC tumor tissues (LC) versus adjacent normal tissues (NLC) based on RNA-seq data. B. Volcano plot displaying the distribution of differentially expressed genes (DEGs), with red and blue dots indicating upregulated and downregulated genes, respectively, and MYBL2 specifically highlighted. C. Venn diagram showing the intersection of DEGs identified from RNA-seq data, the GSE59102 dataset, the TCGA database, and the TCGA-OS analysis. D. Lollipop chart displaying the Hazard Ratios (HR) of candidate genes derived from univariate Cox regression analysis. The dot size represents the log_2_ fold change (log_2_FC) between tumor and normal tissues, while the color gradient indicates the prognostic significance (–log_10_ *P*-value). MYBL2 exhibited the highest risk (HR = 4.99). E. Kaplan-Meier survival curves comparing overall survival (OS) between LSCC patients with high and low MYBL2 expression (HR = 4.99, 95% CI: 2.21−11.27, *P* < 0.01). F. Kaplan-Meier survival curves comparing progression-free survival (PFS) between LSCC patients with high and low MYBL2 expression (HR = 3.17, 95% CI: 1.41−7.13, *P* < 0.01). G. Representative hematoxylin and eosin (H&E) staining showing histological features of adjacent normal tissues, stage II cancer tissues, and stage IV cancer tissues (scale bars: 100 μm and 50 μm). H. Representative immunohistochemistry (IHC) staining showing MYBL2 expression in adjacent normal tissues, stage II cancer tissues, and stage IV cancer tissues (scale bars: 50 μm and 20 μm). I. Gene Set Enrichment Analysis (GSEA) showing enrichment of the GLYCOLYSIS pathway in LSCC samples with high MYBL2 expression (NES = 1.8055, *P*-value < 0.001, FDR = 0.1241). Key leading-edge genes driving the enrichment (*PFKL, GPI, ENO1, PKM, HK1, GAPDH, HK2*) are annotated on the curve. J. Analysis of TCGA-HNSC cohort transcriptome data showing differential expression of MYBL2 between tumor and normal tissues (Wilcoxon test, ***P < 0.001). K. qRT-PCR analysis of MYBL2 mRNA expression levels in human primary laryngeal mucosa epithelial cells (HPLMEC) and various LSCC cell lines. Data are presented as mean ± SEM (**P < 0.01, ***P < 0.001, ns: not significant).

To screen for potential key genes involved in LSCC progression, we implemented an integrative multi-step screening strategy. Initial differential expression analysis identified DEGs between tumor and adjacent normal tissues (Figure 1A, heatmap), with the volcano plot specifically highlighting MYBL2 as a significantly upregulated gene (Figure 1B, ***Supplementary Table S1***). To further narrow down the candidate gene list, we intersected these DEGs with the GSE59102 dataset, TCGA database, and survival-associated gene sets, yielding 30 common candidate genes (Figure 1C, ***Supplementary Table S2***). We then performed univariate Cox regression on these candidates to evaluate their prognostic power. As shown in the prognostic risk ranking profile, MYBL2 emerged as the most promising target, exhibiting the highest hazard ratio (HR = 4.99) and significant prognostic value (Figure 1D). Subsequent Kaplan-Meier survival analysis demonstrated the prognostic significance of MYBL2, revealing that high MYBL2 expression was significantly associated with shorter overall survival (OS) and progression-free survival (PFS) in LSCC patients (Figure 1E–F). Histological staining of LSCC tissue microarrays (TMA) revealed tumor pathology by H&E (Figure 1G) and elevated MYBL2 protein expression by immunohistochemistry (Figure 1H), with expression levels showing a stage-dependent increase. Further statistical analysis of TMA confirmed significant positive correlations between MYBL2 expression levels and T Infiltrate (T stage), lymph node metastasis (*N* stage), and overall clinical stage (Tables 1–3), indicating that upregulated MYBL2 is closely associated with LSCC progression and clinical aggressiveness. Notably, GSEA indicated significant enrichment of the glycolytic pathways in tumors with high MYBL2 expression (Figure 1I), suggesting MYBL2's involvement in glycolytic metabolic reprogramming. Consistent with our TMA findings, MYBL2 mRNA was significantly upregulated in tumor tissues compared to normal controls in the TCGA-LSCC cohort (Figure 1J). Moreover, MYBL2 mRNA levels were predominantly upregulated across diverse LSCC cell lines relative to HPLMEC (Figure 1K), providing a suitable cellular system for subsequent functional investigations.

**Table 1.** Immunohistochemical analysis revealed distinct expression patterns between LSCC and adjacent normal tissues.

MYBL2 expression	LSCC tissues		Adjacent normal tissues		*P* value
	Cases	Percentage	Cases	Percentage	
Low	51	53.1%	24	96%	***P*** **<0.001**
High	45	46.9%	1	4%	

**Table 2.** Association of MYBL2 expression with clinicopathological features in LSCC patients.

Features	No. of patients	MYBL2 (Low)	MYBL2 (High)	*P* value
All patients	96	51	45	
**Gender**				0.318
Male	90	49	41	
Female	6	2	4	
**Histopathological Grade**				0.224
I (well)	31	21	10	
II (Moderate)	50	21	29	
III (Poor)	15	9	6	
**Infiltrate (T)**				***P*** **＜0.001**
T1	12	11	1	
T2	38	25	13	
T3	18	5	13	
T4	28	10	18	
**Lymph node metastasis (*N*)**				***P*** **＜0.001**
N0	67	44	23	
N1	22	6	16	
N2	7	1	6	
**Stage**				***P*** **＜0.001**
I	13	12	1	
II	27	23	4	
III	23	6	17	
IV	33	10	23	

**Table 3.** Spearman's correlation analysis of MYBL2 expression with significant clinicopathological parameters in LSCC.

Variables	Spearman's Correlation (two-tailed)	MYBL2
T Infiltrate (T)	0.390	***P*** **＜0.001**
Lymphatic metastasis (*N*)	0.387	***P*** **＜0.001**
Stage	0.515	***P*** **＜0.001**

### 3.2. MYBL2 promotes malignant phenotypes of LSCC

**Figure 2.**
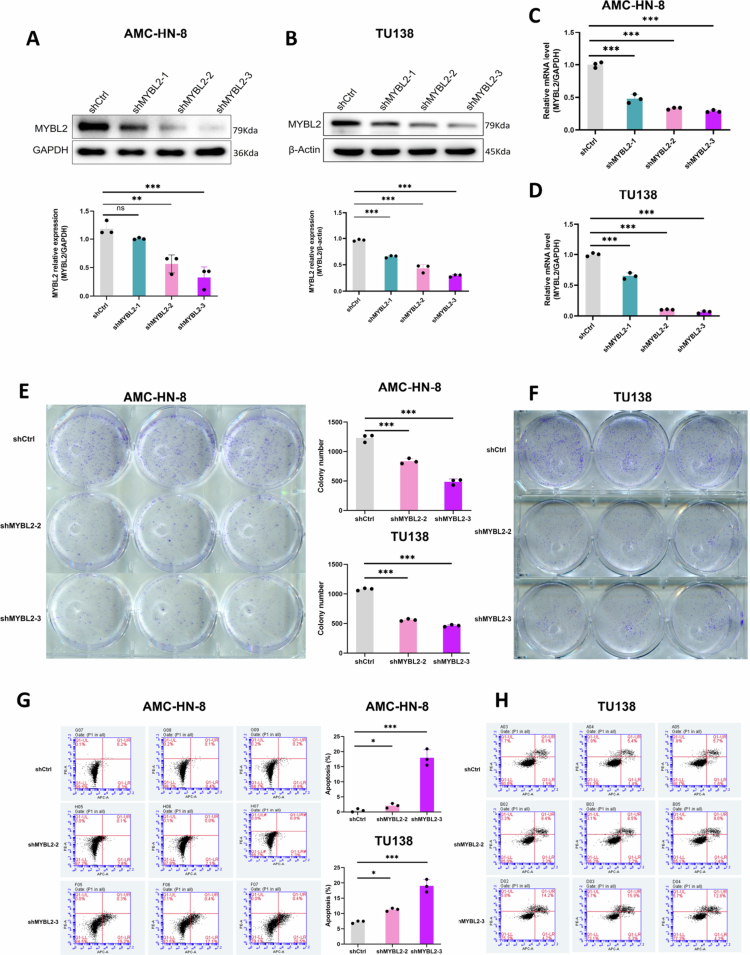
Stable MYBL2 Knockdown Inhibits Invasion and Promotes Apoptosis in LSCC. A–B. Knockdown efficiency confirmation by Western blot after transduction of shMYBL2-1, shMYBL2-2 and shMYBL2-3 in AMC-HN-8 and TU138 cells. Quantification of protein expression is shown below. C–D. Knockdown efficiency confirmation by qRT-PCR after transduction of shMYBL2-1, shMYBL2-2 and shMYBL2-3 in AMC-HN-8 and TU138 cells. E–F. Colony formation assays demonstrating the clonogenic potential of AMC-HN-8 and TU138 cells transduced with shMYBL2-2 and shMYBL2-3. G–H. Flow cytometry analysis of apoptosis rates in AMC-HN-8 and TU138 cells transduced with shMYBL2-2 and shMYBL2-3. Data are presented as mean ± SEM, *n* = 3. *P < 0.05, **P < 0.01, ***P < 0.001.

**Figure 3.**
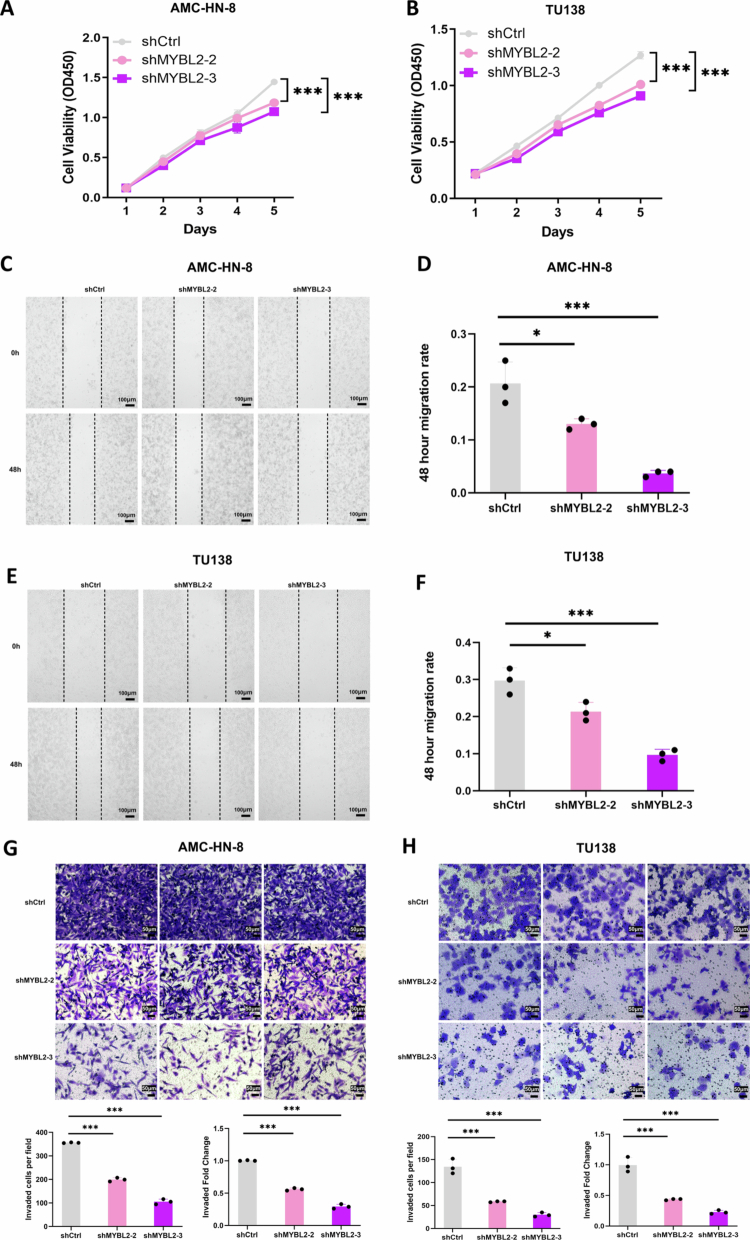
MYBL2 Knockdown Suppresses Proliferation, Migration, and Colony Formation in LSCC. A–B: Cell proliferation analysis using CCK-8 assays in AMC-HN-8 and TU138 cells transduced with shMYBL2-2 and shMYBL2-3. C–F: Wound healing assays showing migration capability of AMC-HN-8 (C, D) and TU138 (E, F) cells transduced with shMYBL2-2 and shMYBL2-3 (scale bar, 100 μm). G–H: Transwell invasion assays showing the invasive capacity of AMC-HN-8 and TU138 cells transduced with shMYBL2-2 and shMYBL2-3 (scale bar, 50 μm). Data are presented as mean ± SEM, *n* = 3. *P < 0.05, **P < 0.01, ***P < 0.001.

To elucidate the biological functions of MYBL2 in LSCC, we established stable MYBL2-knockdown models in AMC-HN-8 and TU138 cell lines. Western blot and qRT-PCR analyzes confirmed significant downregulation of MYBL2 expression at both protein and mRNA levels (Figure 2A–D). Functionally, MYBL2 knockdown reduced long-term colony formation (Figure 2E–F) and increased apoptosis rates (Figure 2G–H), with TU138 cells displaying a more pronounced response than AMC-HN-8 cells. CCK-8 assays further demonstrated decreased cell viability over 5 days (Figure 3A–B). Furthermore, MYBL2 knockdown impaired cell migration in wound healing assays (Figure 3C–F) and reduced invasive capacity in Transwell assays (Figure 3G–H). To validate these findings in a cell line with low endogenous MYBL2 expression, we performed parallel experiments in TU686 cells using colony formation and wound healing assays. Consistent with the results in AMC-HN-8 and TU138 cells, MYBL2 knockdown significantly reduced colony formation and impaired cell migration in TU686 cells (***Supplementary File 1. Fig. S5A–F***). Collectively, these data demonstrate that MYBL2 promotes malignant phenotypes of LSCC cells across multiple cell lines with varying basal expression levels.

### 3.3. MYBL2 drives LSCC progression through metabolic reprogramming of glycolysis

**Figure 4.**
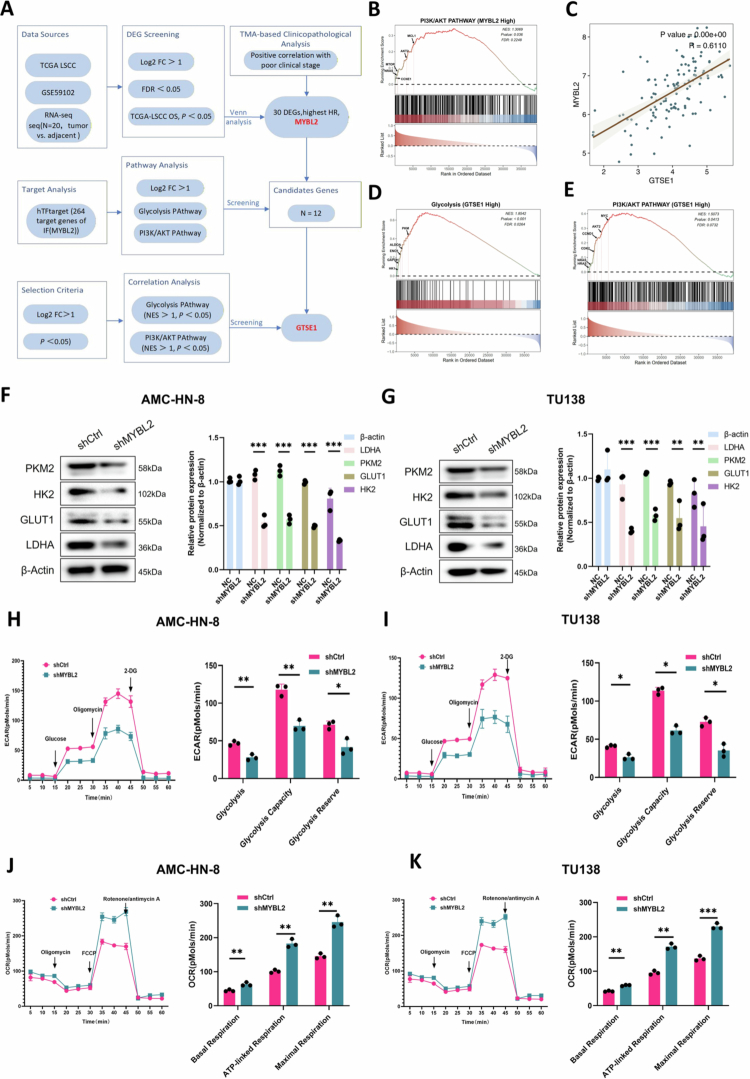
MYBL2 Promotes LSCC Metabolic Reprogramming by Upregulating Glycolytic Proteins. A. Integrated Pipeline for Key Gene Identification and Pathway Enrichment Analysis in LSCC. B. GSEA reveals significant enrichment of PI3K/AKT pathway in MYBL2-high LSCC samples (NES = 1.3069, *P* < 0.01, FDR = 0.2248). Leading-edge genes contributing to the enrichment (*CCNE1, NRAS, MTOR, AKT2, MCL1*) are annotated. C. Correlation analysis between MYBL2 and GTSE1 expression based on the TCGA-LSCC database (*R* = 0.6110, *P* < 0.001). D. GSEA demonstrates enrichment of Glycolysis in GTSE1-high LSCC samples (NES = 1.8542, *P* < 0.001, FDR = 0.0264). Leading-edge genes contributing to the enrichment (*HK1, GAPDH, ENO1, ALDO1, PKM*) are annotated. E. GSEA shows enrichment of PI3K/AKT pathway in GTSE1-high LSCC samples (NES = 1.5073, *P* = 0.0413, FDR = 0.0732). Leading-edge genes contributing to the enrichment (*HRAS, NRAS, CDK2, CCND1, AKT2, MYC*) are annotated. F–G. Western blot analysis of glycolytic proteins (PKM2, HK2, GLUT1, and LDHA) in AMC-HN-8 and TU138 cells transduced with shMYBL2. H–I. Extracellular flux analysis showing the effect of shMYBL2 transduction on extracellular acidification rate (ECAR) in AMC-HN-8 and TU138 cells, including measurements of basal glycolysis, glycolytic capacity, and glycolytic reserve. J–K. Extracellular flux analysis demonstrating the impact of shMYBL2 transduction on oxygen consumption rate (OCR) in AMC-HN-8 and TU138 cells, including measurements of basal respiration, ATP-coupled respiration, and maximal respiratory capacity. Data are presented as mean ± SEM, *n* = 3. * *P* < 0.05, **P < 0.01, *** *P* < 0.001.

A comprehensive bioinformatics analysis strategy was established to investigate MYBL2's downstream regulatory network (Figure 4A). By integrating multiple datasets (TCGA-LSCC, GSE59102, and RNA-seq from 20 paired tumor-normal samples) and applying strict screening criteria (|Log_2_ FC| > 1, FDR < 0.05; for OS, *P* < 0.05), we identified 30 differentially expressed genes associated with poor survival, among which MYBL2 exhibited the highest hazard ratio. TMA-based clinicopathological analysis further validated its clinical relevance. Subsequent hTFTarget prediction and pathway analysis revealed GTSE1 as a key downstream target of MYBL2. GSEA revealed that the PI3K/AKT signaling pathway was significantly enriched in MYBL2-high samples (Figure 4B), and MYBL2 expression positively correlated with GTSE1 expression in the TCGA-LSCC cohort (*R* = 0.6110, *P* < 0.001) (Figure 4C). Similarly, glycolysis and the PI3K/AKT signaling pathway were also significantly enriched in GTSE1-high samples (Figure 4D–E). Western blot analysis demonstrated that MYBL2 knockdown decreased the protein expression of key glycolytic proteins, including PKM2, HK2, GLUT1, and LDHA, in both AMC-HN-8 and TU138 cells (Figure 4F–G). Extracellular flux analysis of LSCC cells revealed that MYBL2 knockdown induced a metabolic shift from glycolysis towards oxidative phosphorylation, characterized by significantly attenuated glycolytic parameters (ECAR: including basal glycolysis, glycolytic capacity, and glycolytic reserve; *P* < 0.01) and concurrent enhancement of mitochondrial respiration metrics (OCR: including basal, ATP-linked, and maximal respiratory capacity; *P* < 0.01) (Figure 4H–K). Collectively, these findings demonstrate MYBL2's crucial role in regulating metabolic reprogramming, potentially through activation of the GTSE1-PI3K/AKT signaling axis.

### 3.4. MYBL2 directly activates GTSE1 transcription

**Figure 5.**
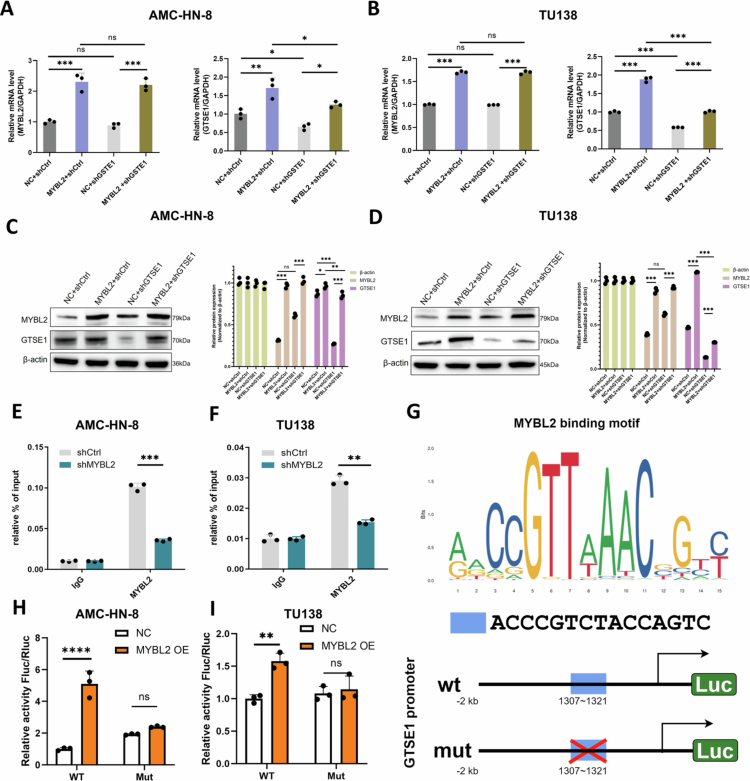
MYBL2 Directly Activates GTSE1 Transcription. A–B. qRT-PCR analysis of GTSE1 mRNA expression in AMC-HN-8 and TU138 cells with MYBL2 overexpression alone or in combination with GTSE1 knockdown. C–D. Western blot analysis of GTSE1 protein levels in AMC-HN-8 and TU138 cells following MYBL2 overexpression alone or combined with GTSE1 knockdown. E–F. ChIP-qPCR analysis of MYBL2 binding to the GTSE1 promoter region in AMC-HN-8 and TU138 cells. G. Bioinformatic prediction of potential MYBL2 binding motif (ACCCGTCTACCAGTC) within the GTSE1 promoter region. H–I. Dual-luciferase reporter assays measuring the effect of MYBL2 overexpression on wild-type and mutant GTSE1 promoter activities in AMC-HN-8 and TU138 cells. Data are presented as mean ± SE, *n* = 3. *P < 0.05, **P < 0.01, ***P < 0.001, ns: not significant.

We performed molecular analyzes to investigate the regulatory relationship between MYBL2 and GTSE1. In both AMC-HN-8 and TU138 cells, MYBL2 overexpression significantly increased GTSE1 expression at both the mRNA and protein levels (Figure 5A–D). The direct binding of MYBL2 to the GTSE1 promoter was demonstrated by ChIP-qPCR experiments (Figure 5E–F). Furthermore, bioinformatics analysis identified a specific MYBL2 binding motif (ACCCGTCTACCAGTC) located in the GTSE1 promoter region (Figure 5G). The functional significance of this binding was validated using dual-luciferase reporter assays. MYBL2 overexpression significantly enhanced wild-type GTSE1 promoter activity but had no effect on the mutant promoter (Figure 5H–I), confirming that MYBL2 directly activates GTSE1 transcription.

### 3.5. MYBL2 regulates LSCC malignant phenotypes via GTSE1

**Figure 6.**
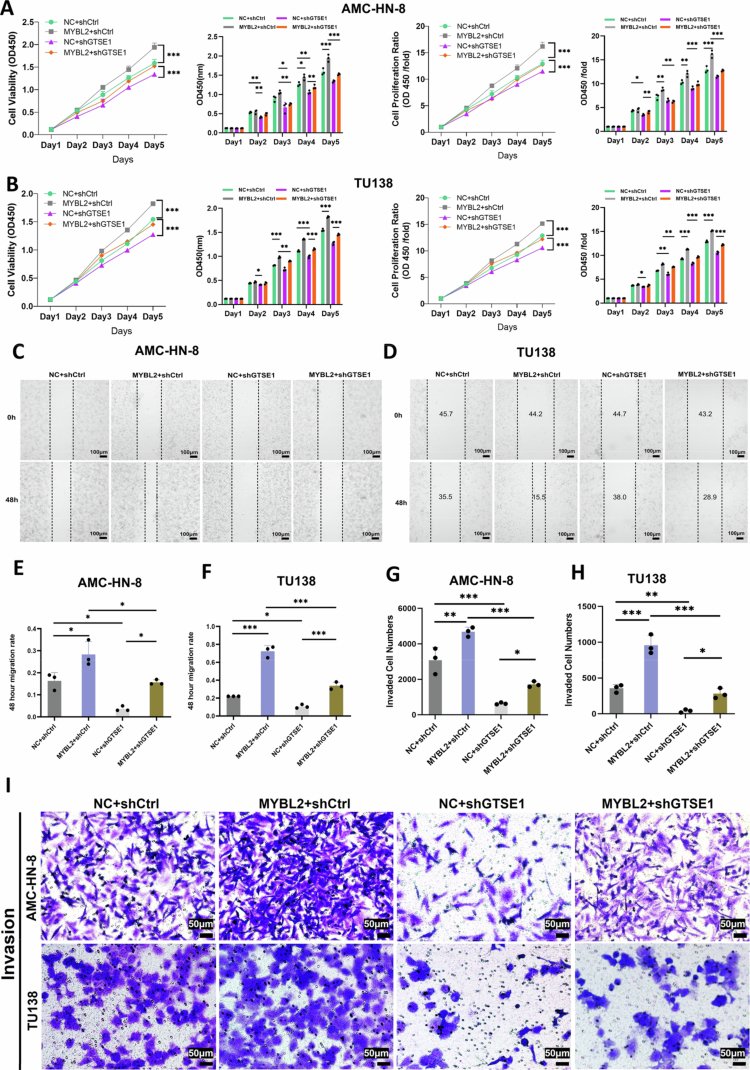
The MYBL2-GTSE1 Regulatory Axis Promotes Cell Proliferation, Migration, and Invasion. A–B. CCK-8 assays showing the effects of MYBL2 overexpression alone or combined with GTSE1 knockdown on AMC-HN-8 and TU138 cell proliferation. C–D. Wound healing assays demonstrating the impact of MYBL2 overexpression alone or combined with GTSE1 knockdown on AMC-HN-8 and TU138 cell migration (scale bar, 100 μm). E–F. Quantitative analysis of cell migration from panels C–D. G–H. Quantitative analysis of 48 h invasion rates from panel I. I. Transwell invasion assays evaluating the effects of MYBL2 overexpression alone or combined with GTSE1 knockdown on AMC-HN-8 and TU138 cell invasion (scale bar, 50 μm). Data are presented as mean ± SEM, *n* = 3. *P < 0.05, **P < 0.01, ***P < 0.001.

To elucidate the functional necessity of GTSE1 in mediating the oncogenic effects of MYBL2, rescue experiments were performed. Using shGTSE1 validated for its knockdown efficacy ***(Supplementary File 1. Fig. S6A–E)***, we found that in both AMC-HN-8 and TU138 cells, the enhanced cell proliferation induced by MYBL2 overexpression was significantly attenuated by GTSE1 knockdown, as demonstrated by CCK-8 assays (Figure 6A–B). Similarly, wound healing assays showed that GTSE1 knockdown significantly reduced MYBL2-mediated cell migration (Figure 6C–D, quantified in Figure 6E–F). Transwell invasion assays further showed that GTSE1 knockdown effectively suppressed MYBL2-induced invasion (Figure 6I, quantified in Figure 6G–H). Collectively, these results demonstrate that GTSE1 serves as an essential mediator of MYBL2-induced proliferative, migratory, and invasive phenotypes.

### 3.6. MYBL2-GTSE1 axis regulates glycolysis through PI3K/AKT signaling

**Figure 7.**
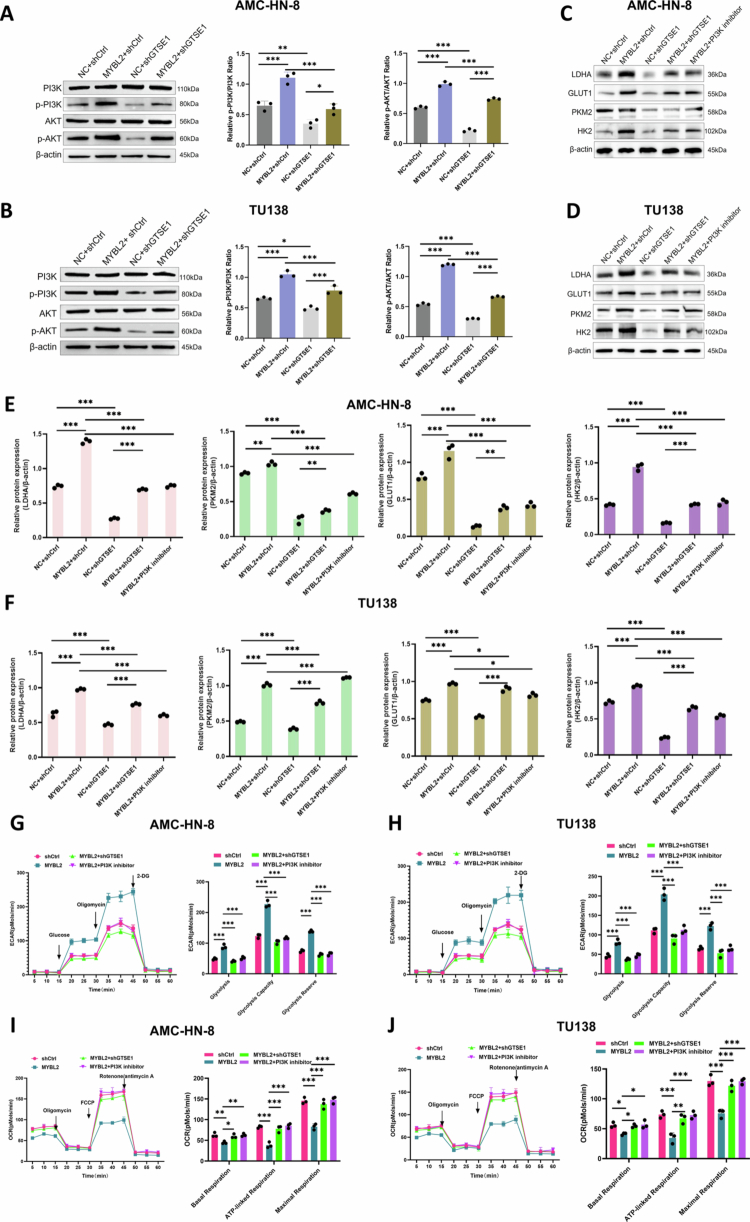
MYBL2-GTSE1 Axis Activates PI3K/AKT Signaling and Regulates Glycolysis. A–B. Western blot analysis of *p*-PI3K and *p*-AKT levels in AMC-HN-8 and TU138 cells with MYBL2 overexpression alone or combined with GTSE1 knockdown. The right panels show quantification of relative protein expression. C–D. Western blot analysis of glycolytic proteins (LDHA, GLUT1, PKM2, HK2) in AMC-HN-8 and TU138 cells following MYBL2 overexpression alone or combined with GTSE1 knockdown/PI3K inhibitor (LY294002, 10 μM, 24 h) treatment. E–F. Quantitative analysis of glycolytic protein expression from panels C–D. G–H. ECAR analysis showing glycolytic capacity in AMC-HN-8 and TU138 cells under MYBL2 overexpression alone or combined with GTSE1 knockdown/PI3K inhibitor treatment. I–J. OCR analysis demonstrating respiratory function in AMC-HN-8 and TU138 cells under MYBL2 overexpression alone or combined with GTSE1 knockdown/PI3K inhibitor treatment. Data are presented as mean ± SEM, *n* = 3. *P < 0.05, **P < 0.01, ***P < 0.001.

To validate the predicted involvement of PI3K/AKT signaling in the MYBL2-GTSE1 axis, we performed functional experiments. Western blot analysis showed that MYBL2 overexpression increased PI3K and AKT phosphorylation, which was attenuated by GTSE1 knockdown (Figure 7A–B). We further examined key glycolytic proteins and found that MYBL2 overexpression significantly increased LDHA, GLUT1, PKM2, and HK2 protein levels. Either GTSE1 knockdown or PI3K inhibitor (LY294002) treatment attenuated these increases (Figure 7C–F). Similar results were observed with AKT inhibitor (MK-2206) treatment (***Supplementary File1. Fig. S3A–B***). Extracellular flux analysis showed that both GTSE1 knockdown and PI3K inhibition attenuated the MYBL2-induced metabolic changes. Specifically, MYBL2 overexpression increased glycolysis (elevated ECAR) and decreased oxidative phosphorylation (reduced OCR), and these effects were reversed by GTSE1 knockdown or PI3K inhibition (Figure 7G–J). These findings demonstrate a signaling axis in which MYBL2 transcriptionally activates GTSE1, which then increases PI3K/AKT phosphorylation. This leads to upregulation of key glycolytic proteins and promotes a glycolytic metabolic phenotype in LSCC cells.

### 3.7. GTSE1 knockdown reserved MYBL2-induced tumor growth *In Vivo*

**Figure 8.**
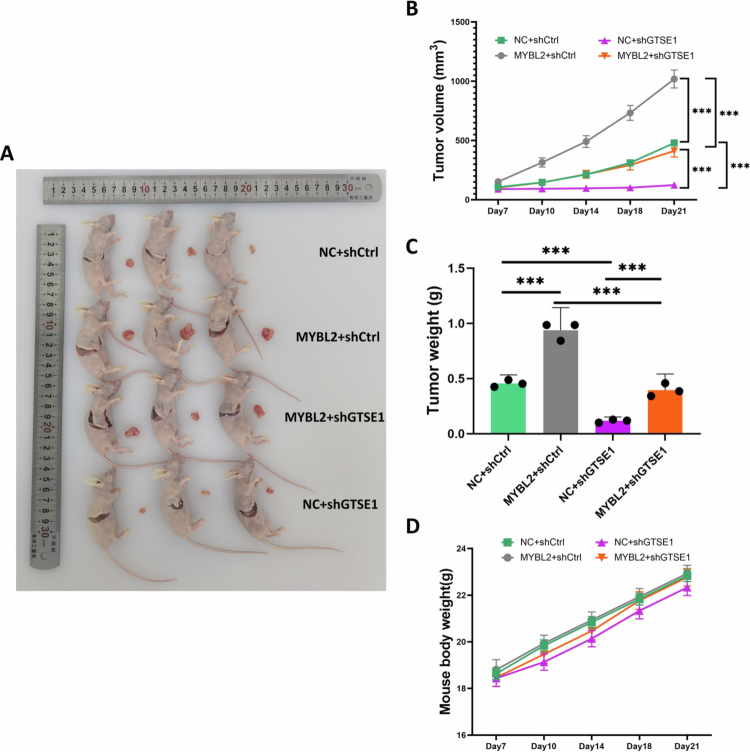
GTSE1 Knockdown Reversed MYBL2-Induced Tumor Growth *In Vivo*. A–C. Subcutaneous xenograft tumor model with MYBL2 overexpression alone or combined with GTSE1 knockdown. Representative tumor images (A), tumor volume growth curves (B), and final tumor weights (C) are shown. D. Body weight monitoring throughout the experimental period. Data are presented as mean ± SEM, *n* = 3. ***P < 0.001.

To substantiate the oncogenic role of the MYBL2-GTSE1 axis in *vivo*, we established a subcutaneous xenograft tumor model in nude mice. MYBL2 overexpression significantly promoted tumor growth, an effect that was effectively reversed by GTSE1 knockdown, as demonstrated by both macroscopic observation and longitudinal tumor volume measurements (Figure 8A–B). Final tumor weights further confirmed these observations (Figure 8C). No adverse effects on mouse body weight were observed throughout the study period (Figure 8D). These *in vivo* data demonstrate that the MYBL2-GTSE1 regulatory axis plays a functional role in tumor development.

## 4. Discussion

Metabolic reprogramming has become a critical focus in cancer research over the past decade.^37^ Accordingly, strategies targeting key molecular regulators of these metabolic pathways have demonstrated significant therapeutic potential.^38^ Preclinical studies have demonstrated significant antitumor activity with various glycolytic inhibitors, including BAY-876 (GLUT1 inhibitor),^39^ 2-deoxyglucose (HK2 inhibitor),^40^ and FX11 (LDHA inhibitor).^41^ Moreover, the AZD3965 (MCT1/4 inhibitor) has progressed to clinical trials, underscoring the clinical relevance of targeting lactate efflux.^42^ Similar to other solid tumors, LSCC displays elevated glycolysis to meet its metabolic demands, suggesting a close association between glycolysis and LSCC pathogenesis and progression.^43^^,^^44^ In this study, we identify a novel molecular axis contributing to this metabolic reprogramming. Initiating from the clinical observation that MYBL2 is upregulated in LSCC and correlates with poor prognosis, we demonstrated that MYBL2 promotes glycolysis by transcriptionally activating GTSE1, which subsequently activates the PI3K/AKT signaling pathway. Crucially, these mechanistic findings were substantiated by *in vivo* xenograft tumor models, where GTSE1 knockdown effectively reversed MYBL2-driven tumor growth. Collectively, our study identifies the MYBL2-GTSE1 axis as an important contributor to glycolytic reprogramming in LSCC.

Analysis across multiple databases demonstrated elevated MYBL2 expression in LSCC and its correlation with unfavorable clinical outcomes, a pattern that aligns well with previous observations in several other cancer types.^45-47^ As a transcription factor, MYBL2 has been previously shown to directly bind and transcriptionally activate downstream target genes, including cell division cycle-associated 3 (CDCA3) in bladder cancer ^47^ and actin-binding Rho-activating protein C-terminal like protein (ABRACL) in breast cancer,^48^ thereby driving malignant phenotypes. Consistent with previous reports in other cancer types, our functional studies showed that MYBL2 knockdown significantly inhibited proliferation, migration, and invasion while inducing apoptosis across both LSCC cell lines, but with some variation in response magnitude. Moreover, MYBL2 regulates cell proliferation and differentiation by modulating cell cycle-related genes, including CDK2, c-MYC, and Cyclin D2.^49^ Notably, our GSEA analysis revealed a significant correlation between MYBL2 overexpression and glycolysis pathway activation in LSCC, which was subsequently validated by experimental studies demonstrating MYBL2's role in promoting glycolysis. These findings suggest a novel role for MYBL2 in metabolic reprogramming. Research on metabolic reprogramming in LSCC remains less extensively characterized compared to other Head and Neck Squamous Cell Carcinoma (HNSC) subtypes. While metabolic studies in oral squamous cell carcinoma (OSCC) ^50^^,^^51^ and nasopharyngeal carcinoma (NPC) ^52^^,^^53^ have extensively explored glutaminolysis,^54^ fatty acid synthesis,^55^ and metabolic symbiosis ^56^^,^^57^ within the tumor microenvironment, investigations into LSCC metabolism have largely focused on the Warburg effect, with the underlying regulatory mechanisms remaining incompletely understood.

We further identified and characterized a novel transcriptional relationship between MYBL2 and GTSE1 in LSCC. Using ChIP-qPCR and dual-luciferase reporter assays, we established GTSE1 as a novel direct transcriptional target of MYBL2 in LSCC, thereby expanding MYBL2's known target gene repertoire. GTSE1 has been extensively recognized for its regulatory roles in the cell cycle ^58^ and DNA damage repair.^59^ Recent studies have shown that its overexpression is associated with the progression of various malignancies, including hepatocellular carcinoma,^60^ breast cancer,^61^ and gastric cancer.^62^ However, the role of GTSE1 in tumor metabolic reprogramming remains incompletely characterized.^27^ Functional rescue experiments demonstrated that GTSE1 is not merely a downstream target of MYBL2 but rather serves as an essential functional mediator of its oncogenic properties, including enhanced proliferation and migration. The identification of the MYBL2-GTSE1 axis provides a molecular bridge linking transcriptional regulation to signal transduction and expands the known biological function of GTSE1, and suggests an important role for GTSE1 in tumor metabolic reprogramming.

Bioinformatics predictions and experimental data converged on the PI3K/AKT signaling pathway, a central hub for cellular growth and metabolism. Activation of the PI3K/AKT pathway has been recognized as a key mechanism driving the “Warburg effect” in tumors, by promoting the expression of key glycolytic enzymes and glucose transporters.^25^^,^^63^^,^^64^ Our findings are consistent with this mechanism: MYBL2-induced GTSE1 upregulation significantly enhanced the phosphorylation levels of PI3K and AKT, which in turn increased the expression of key glycolytic enzymes (including HK2, PKM2, LDHA, and GLUT1), ultimately resulting in elevated ECAR and decreased OCR. Notably, GTSE1 knockdown or LY294002(PI3K inhibitor)/MK-2206 (AKT inhibitor) effectively reversed MYBL2-driven glycolysis and tumor progression, demonstrating the functional importance of this signaling axis. Our findings are consistent with previous observations in induced pluripotent stem cells (iPSCs), where LY294002-mediated PI3K/AKT inhibition led to decreased glycolysis and reduced pluripotency factor expression.^65^ Collectively, we have identified a regulatory axis linking transcriptional control to downstream metabolic phenotypes through the PI3K/AKT signaling pathway.

In conclusion, our study demonstrates that MYBL2 regulates LSCC metabolic reprogramming through transcriptional activation of GTSE1 and subsequent activation of the PI3K/AKT pathway. Although immunodeficient xenograft models were used with limited *in vivo* validation, the consistency across *in vitro* mechanistic studies, *in vivo* functional experiments, and clinical tissue analyzes supports the clinical relevance and translational potential of this regulatory axis. Future studies could investigate the molecular mechanisms by which GTSE1 activates PI3K/AKT signaling, including the potential role of post-translational modifications. Collectively, this study characterizes a metabolic regulatory mechanism and suggests the MYBL2-GTSE1 axis as a potential therapeutic target in LSCC.

## Supplementary Material

Supplementary File 3.docx Supplementary File 3.docx

Supplementary File 2.docx Supplementary File 2.docx

Supplementary File 1.docx Supplementary File 1.docx

Supplementary Table S1.xlsx Supplementary Table S1.xlsx

Supplementary Table S2.docx Supplementary Table S2.docx

Author Checklist.pdf Author Checklist.pdf

## Data Availability

The datasets used and/or analyzed during the current study are available from the corresponding author on reasonable request.

## Abbreviations

LSCC: Laryngeal Squamous Cell Carcinoma
HNSC: Head and Neck Squamous Cell Carcinoma
TCGA: The Cancer Genome Atlas
MYBL2: MYB Proto-Oncogene Like 2
GTSE1: G2 and S phase expressed protein 1
DEGs: Differentially expressed genes
GSEA: Gene Set Enrichment Analysis
NES: normalized enrichment score
FDR: false discovery rate
HPLMEC: Human Primary Laryngeal Mucosal Epithelial Cells
SEM: Standard Error of the Mean
GEO: Gene Expression Omnibus
ECAR: Extracellular Acidification Rate
OCR: Oxygen Consumption Rate
TMAs: Tissue microarrays
OS: overall survival
PFS: Progression-Free Survival
