# Supplementary material for: The MYBL2-GTSE1 axis promotes laryngeal squamous cell carcinoma progression by regulating PI3K/AKT-dependent glycolytic reprogramming
Source: Cancer Biol Ther. 2026 Mar 22;27(1):2648193. doi: 10.1080/15384047.2026.2648193 (PMC13011630; doi:10.1080/15384047.2026.2648193)

**Cell Line Authentication Report (STR Profiling)**

**Report Date:** November 08, 2024 **Service Provider:** Biowing Biotechnology Co., Ltd. (Shanghai, China)

**1. Sample Information**

- **Sample Name:** TU138
- **Sample ID:** 20241105-15
- **Methodology:** 21-locus STR (Short Tandem Repeat) multiplex amplification analysis using ABI 3730XL Genetic Analyzer.

**2. Authentication Result**

- **Conclusion:** **MATCH**
- **Matched Cell Line:** **Tu 138** (ExPASy Accession: **CVCL_4910**)
- **Match Score (EV):** **0.96** (An EV ≥ 0.80 indicates the cell lines are related/identical).
- **Status:** No multi-allelic contamination was detected. The profile quality is good.

**3. STR Genotyping Data (Comparison)** The sample STR profile was compared with the reference profile from the **Tu 138** cell line in the database.

| **Loci** | **Sample Profile** | **Note** |
| --- | --- | --- |
| **D5S818** | 12, 12 | ✓ Match |
| **D13S317** | 12, 12 | ✓ Match |
| **D7S820** | 10, 10 | ✓ Match |
| **D16S539** | 8, 9 | ✓ Match |
| **vWA** | 15, 16 | ✓ Match |
| **TH01** | 8, 8 | ✓ Match |
| **AMEL** | X, X | ✓ Match |
| **TPOX** | 10, 10 | *Minor variant (Ref: 10,11)* |
| **CSF1PO** | 12, 13 | ✓ Match |
|  |  |  |
| **Original file** |  |  |


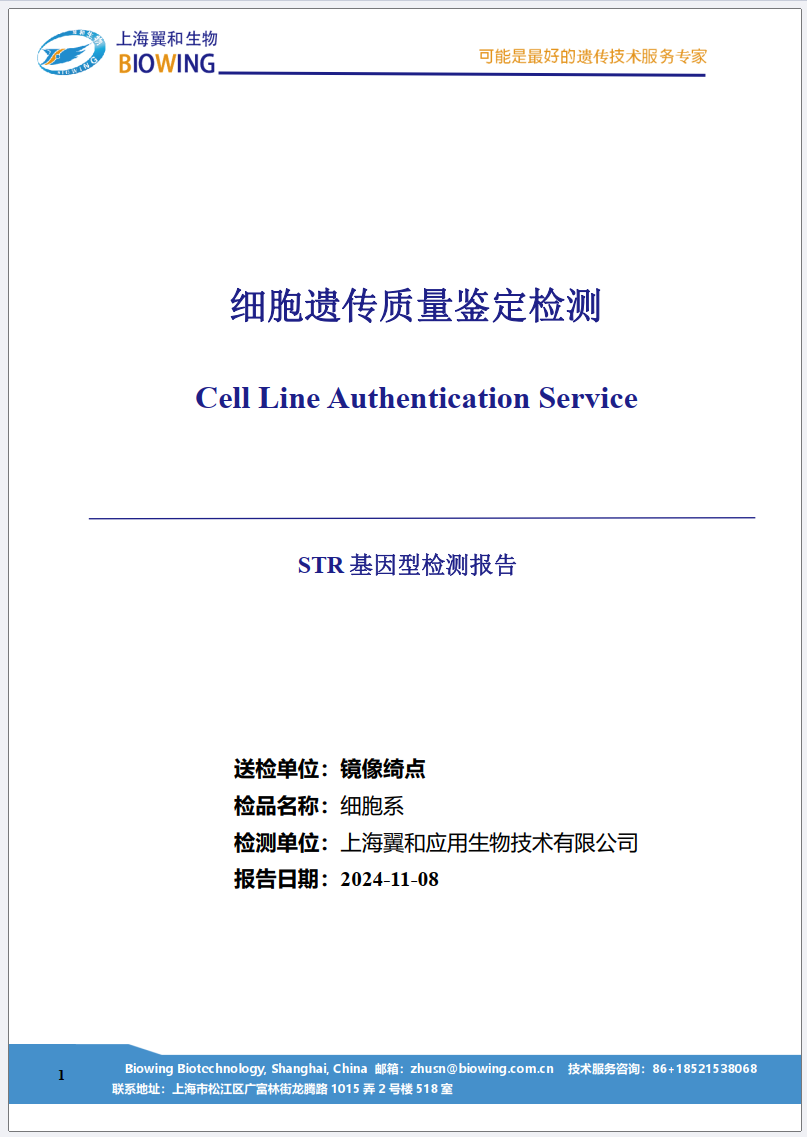

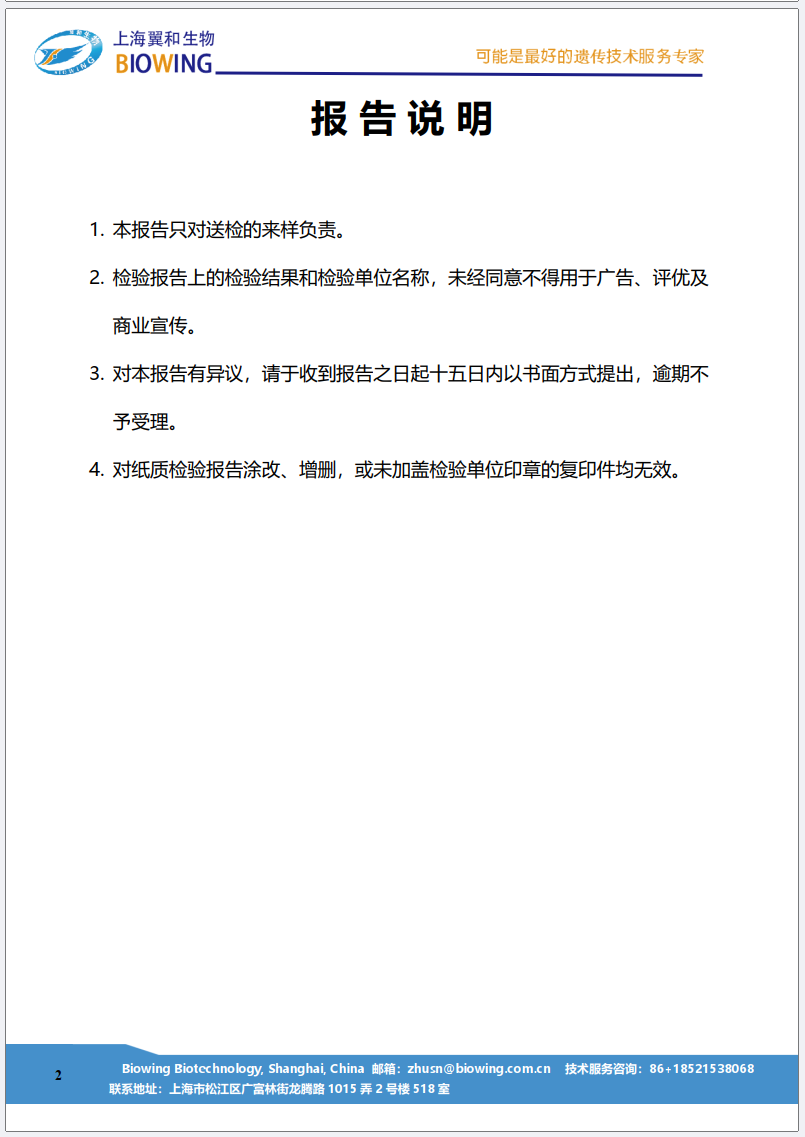

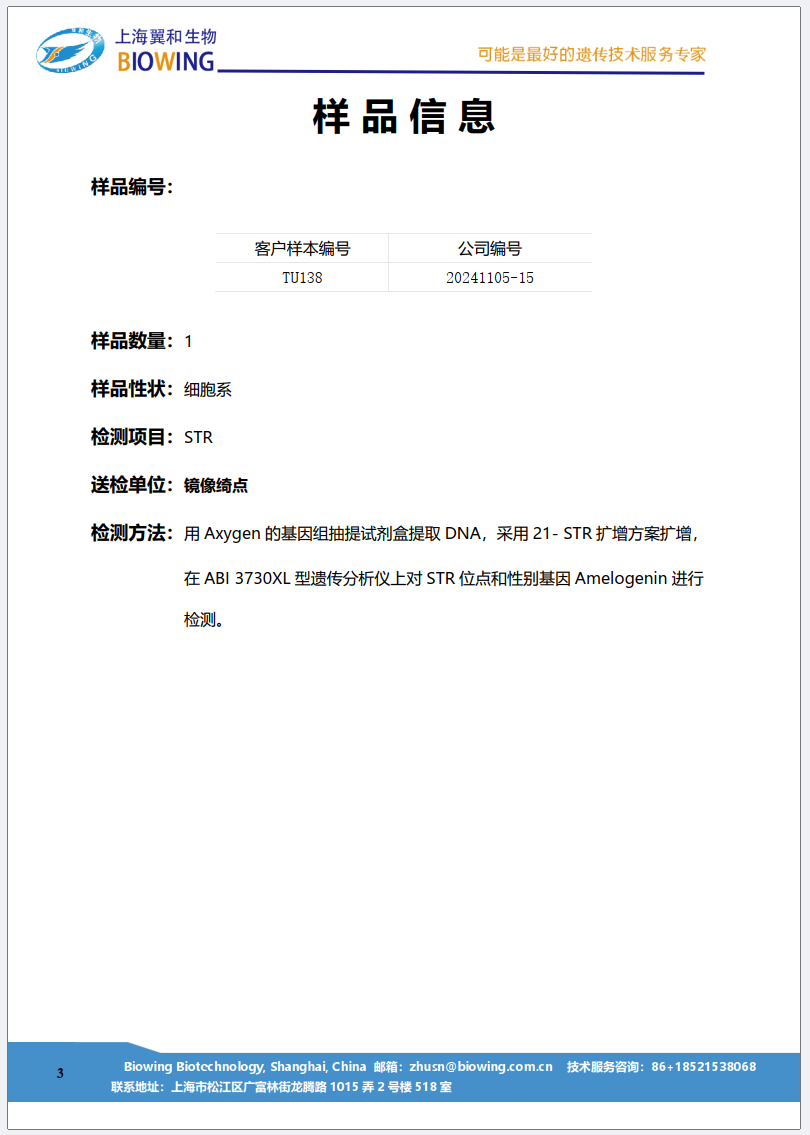

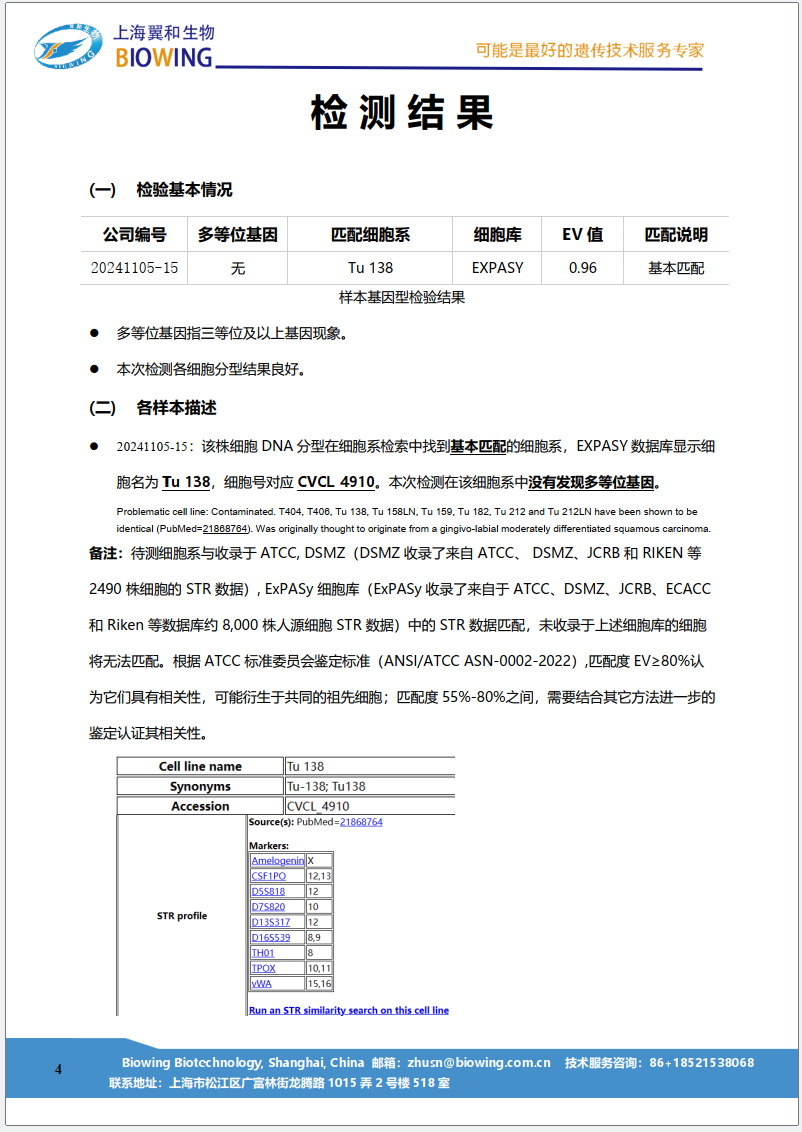

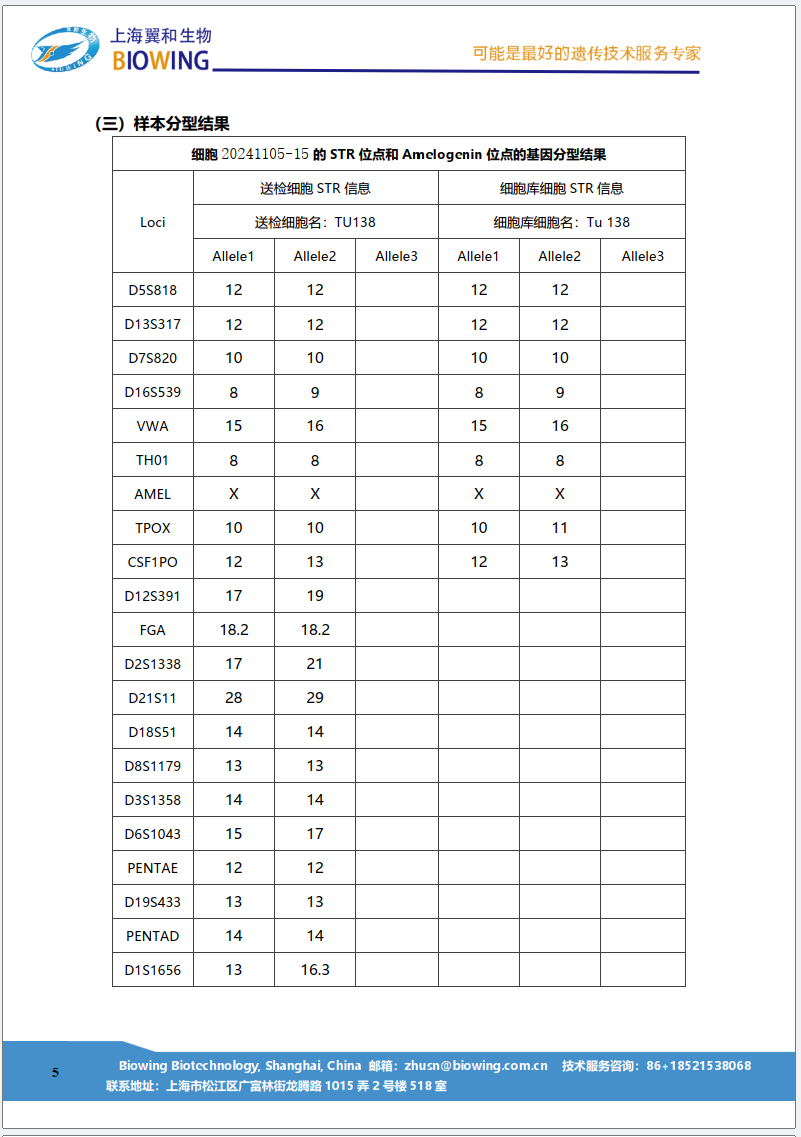

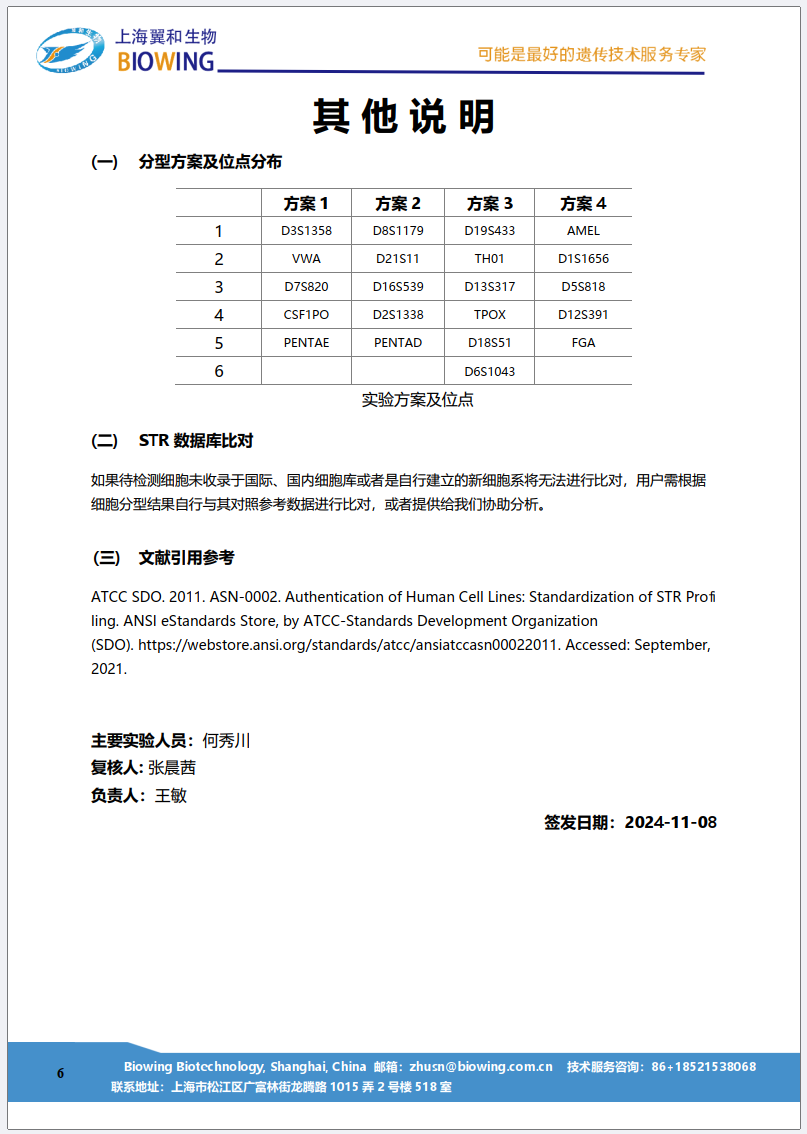

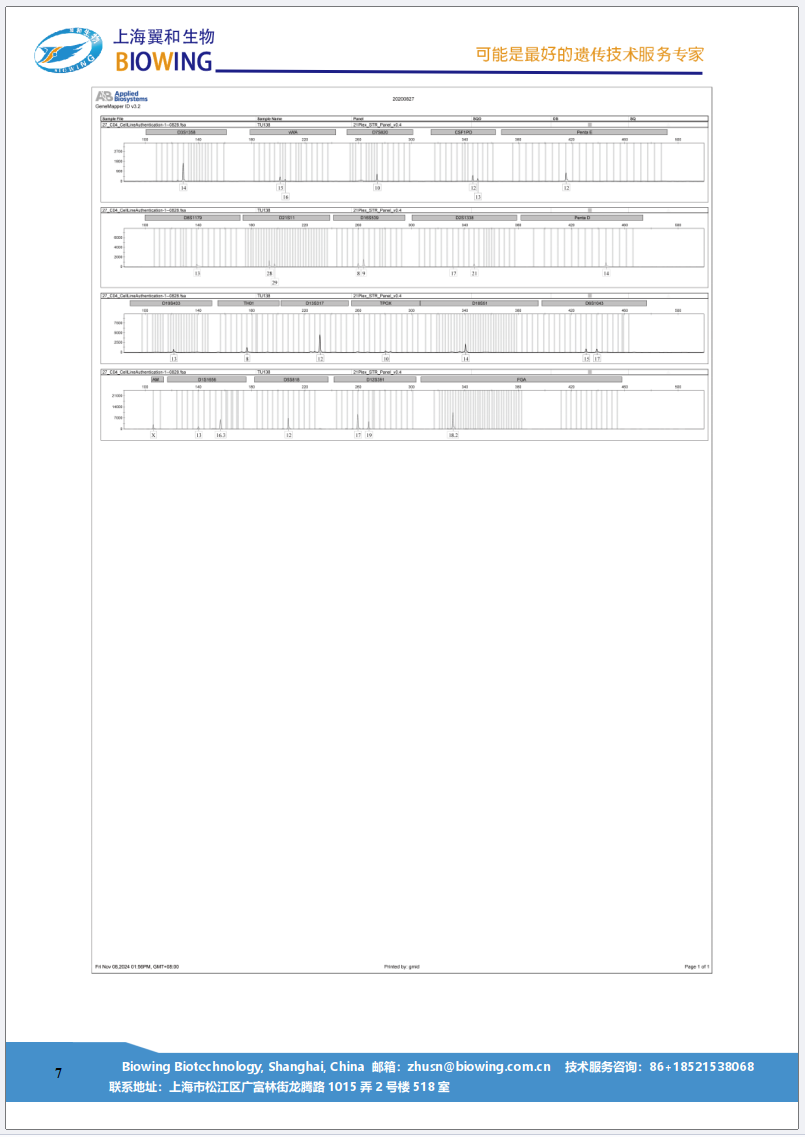


**Mycoplasma Detection Report**

**Testing Company:** Cellecta Biosciences (Shanghai) Co., Ltd.
**Address:** 2F, Building 3, Juke Biopark, 466 Yindu Road, Xuhui District, Shanghai
**Report Date:** April 28, 2025

**Sample Information**

| **No.** | **Cell Line** | **Sample Type** |
| --- | --- | --- |
| **1** | **Human primary arm skin fibroblasts** | **Cell culture** |
| **2** | **TU138** | **Cell culture** |
| **3** | **C2C12** | **Cell culture** |
| **4** | **Mouse embryonic fibroblasts** | **Cell culture** |

**Total Samples:** 4
**Detection Method:** PCR-based mycoplasma detection

**Detection Protocol**

**Sample Preparation**

1. Collect 1 mL cell culture supernatant (cultured >48 hours)
2. Centrifuge at 16,000g for 5 minutes
3. Wash pellet 3× with sterile PBS
4. Resuspend in 100 μL ultrapure water
5. Heat at 100°C for 5 minutes

**PCR Detection**

**PCR Conditions:**

- Initial denaturation: 94°C, 3 min
- Touchdown PCR: 18 cycles (94°C-30s, 68°C-30s, 72°C-30s, -1°C/cycle)
- Standard PCR: 32 cycles (94°C-30s, 60°C-30s, 72°C-30s)
- Final extension: 72°C, 3 min

**Gel Electrophoresis**

- 1.5% agarose gel with GelRed
- 120V, ~20 minutes
- Expected bands: Positive control (700 bp), Mycoplasma positive (500 bp)

**Results**

**PCR Gel Lanes:**

1. Marker | 2. Negative control | 3. Positive control | 4-11. Test samples with internal controls

**Final Results**

**TU138: MYCOPLASMA NEGATIVE**

All four tested cell lines showed **negative results** for mycoplasma contamination.

**Primary Technician:** Wu Kangcheng
**Reviewer:** Zhang Lili
**Supervisor:** Huo Chaochao
**Issue Date:** April 28, 2025

**Cellecta Biosciences (Shanghai) Co., Ltd.**

**Original file**


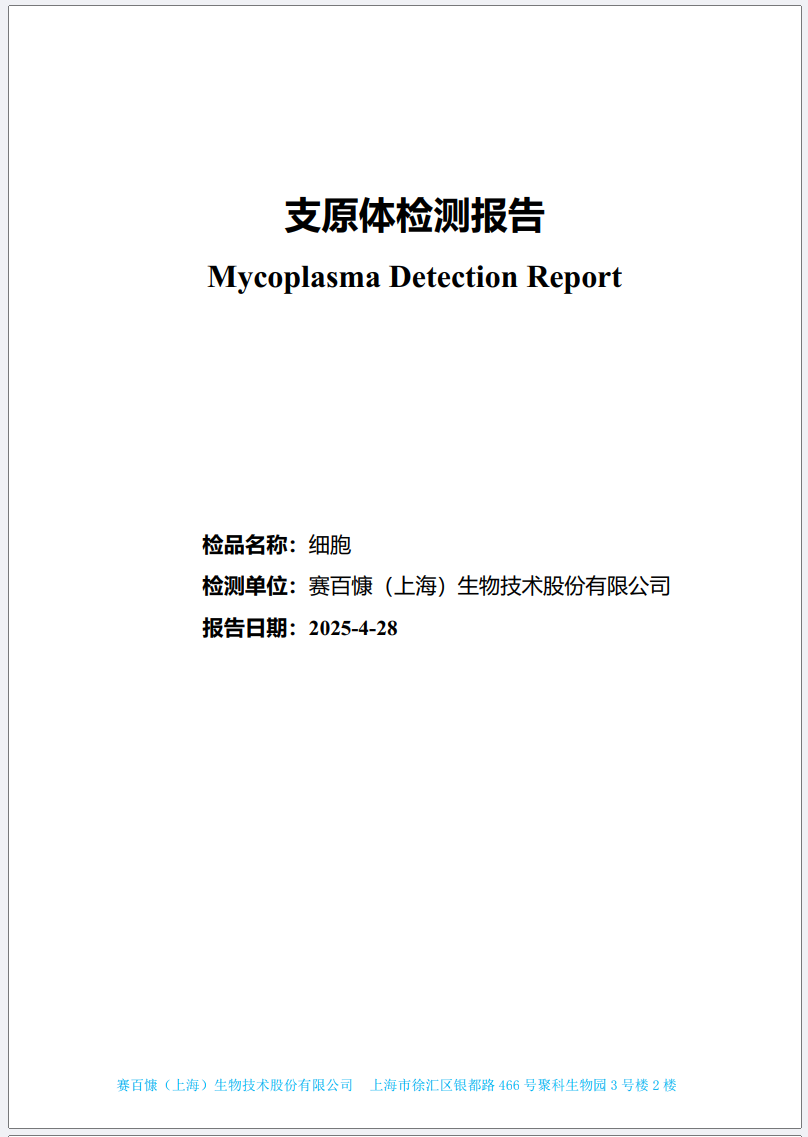

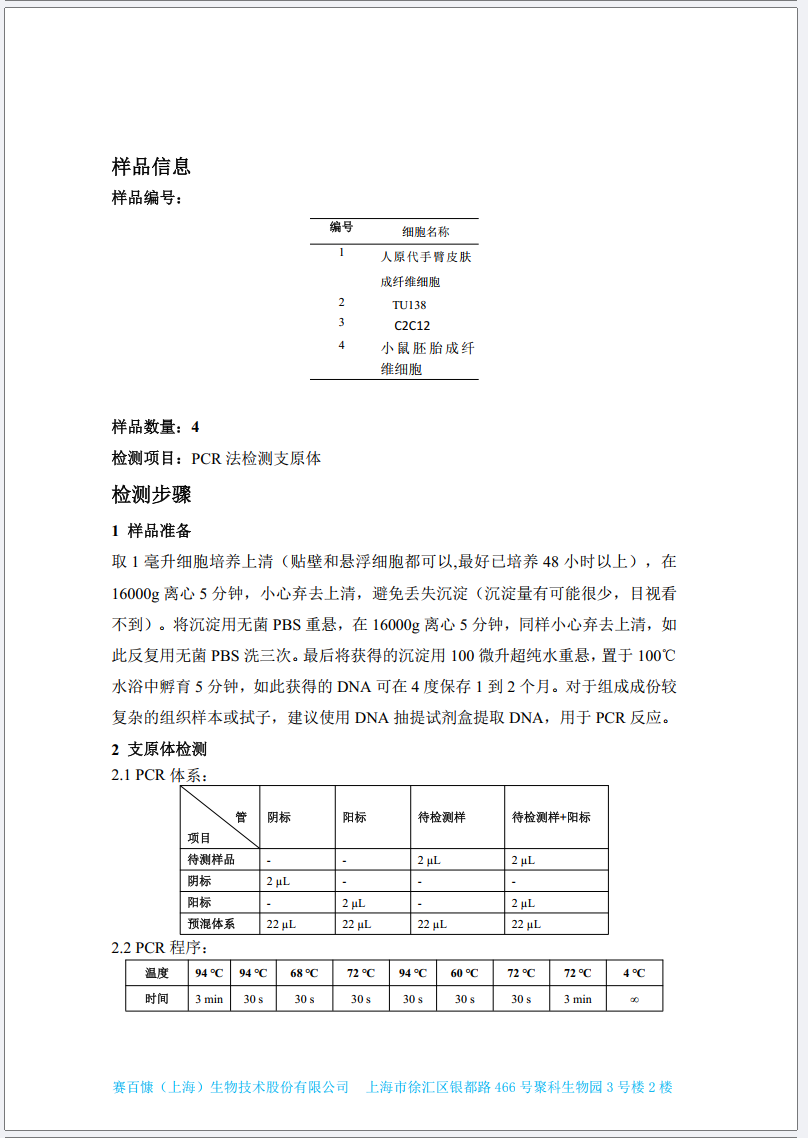

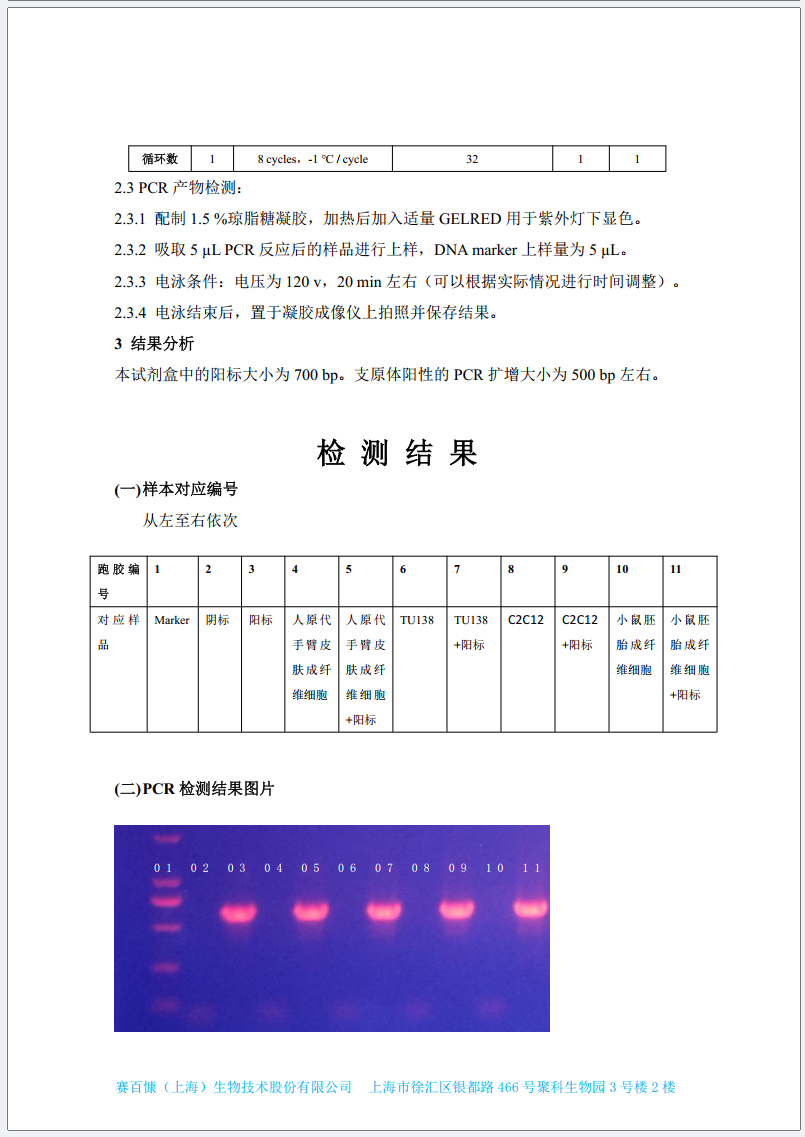

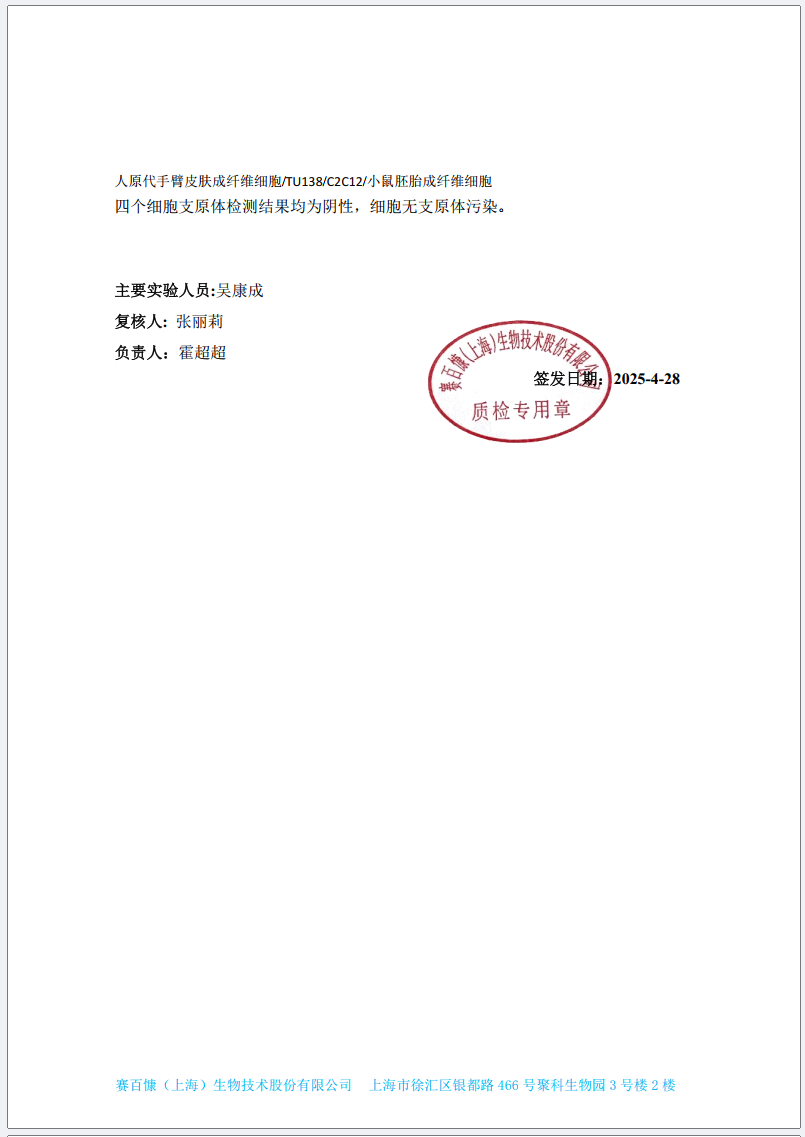


**Original file**


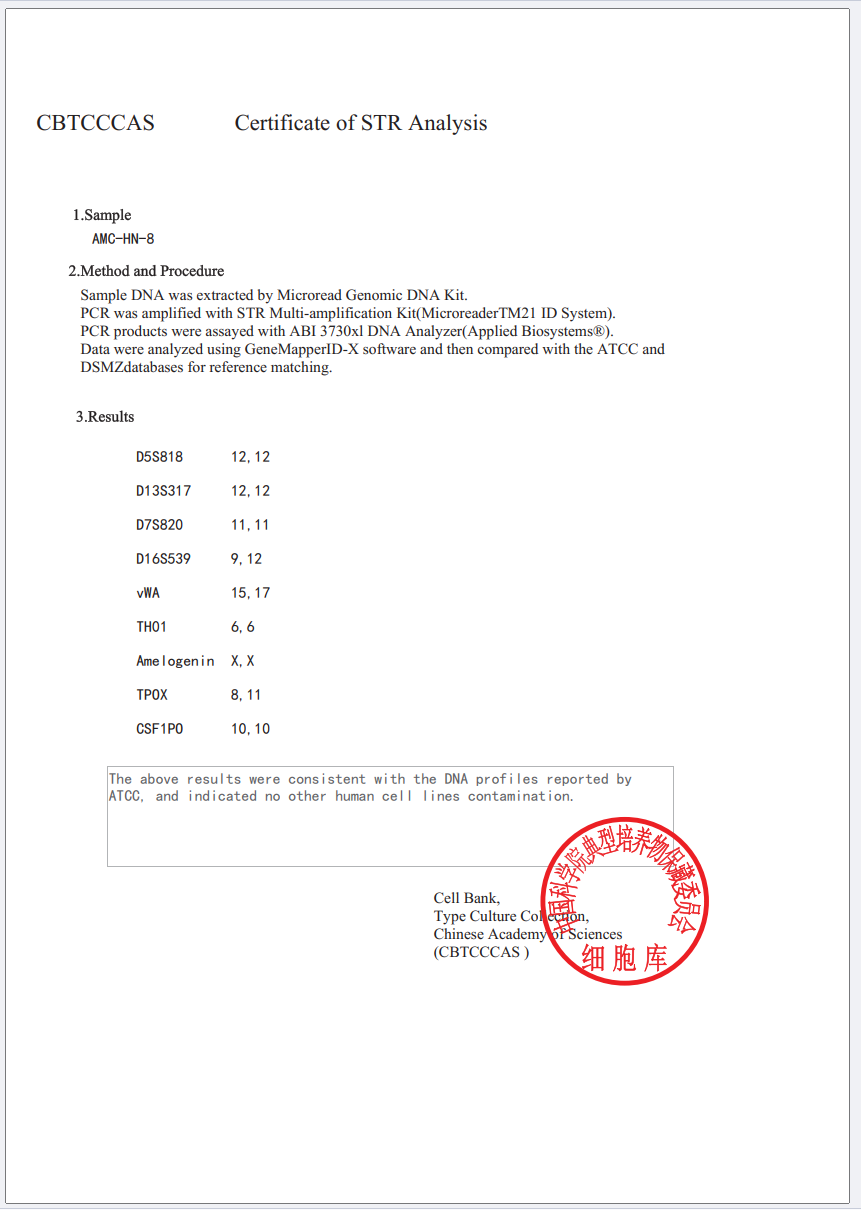


**Certificate of Analysis**

**Issuer:** CBTCCCAS (Cell Bank, Type Culture Collection, Chinese Academy of Sciences)

**Sample** AMC-HN-8

**Quality Profile**

| **Test** | **Method** | **Result** |
| --- | --- | --- |
| **Bacteria** | **Cultured** | **None detected** |
| **Fungi** | **Cultured** | **None detected** |
| **Mycoplasma** | **Cultured** | **None detected** |

**Conclusion:** Conclusion: Quality control testing confirmed that the AMC-HN-8 cell line is free from microbial contamination (bacteria, fungi, and mycoplasma).

**Original file**


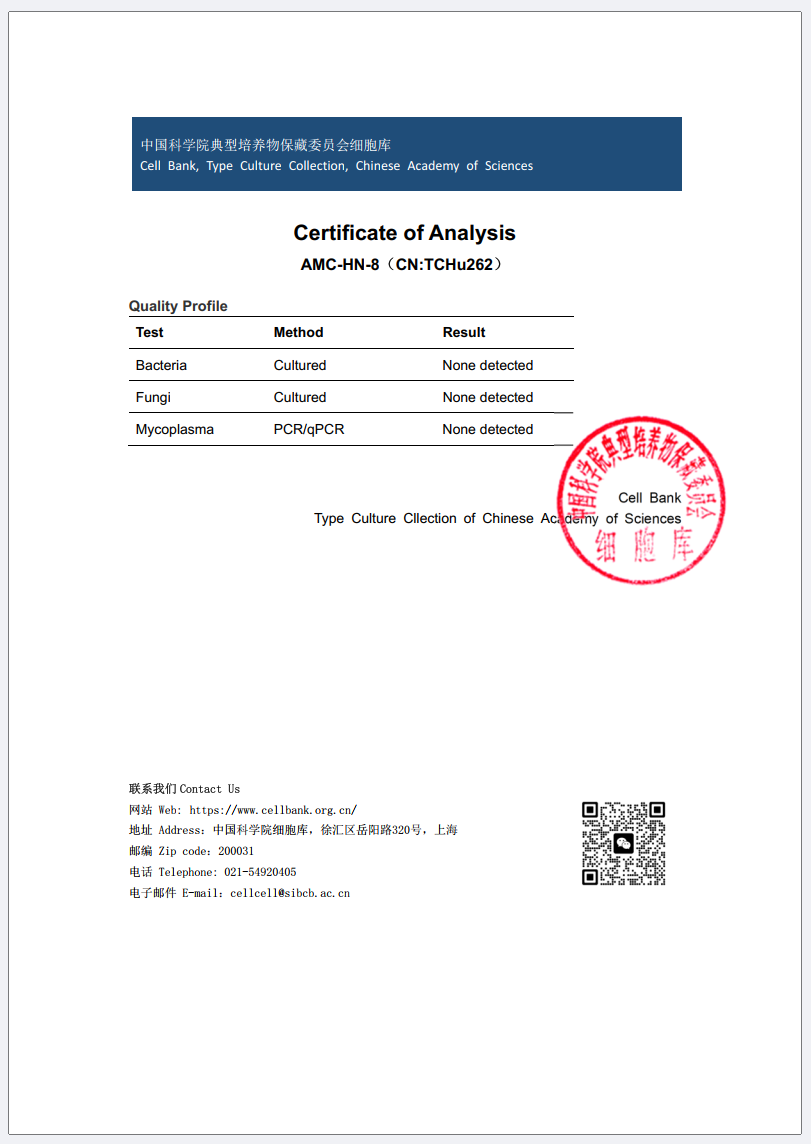

Supplement: Supplementary File 3.docx [file KCBT_A_2648193_SM0571.docx]
