# Supplementary material for: The MYBL2-GTSE1 axis promotes laryngeal squamous cell carcinoma progression by regulating PI3K/AKT-dependent glycolytic reprogramming
Source: Cancer Biol Ther. 2026 Mar 22;27(1):2648193. doi: 10.1080/15384047.2026.2648193 (PMC13011630; doi:10.1080/15384047.2026.2648193)
Supplement: Supplementary File 2.docx [file KCBT_A_2648193_SM0570.docx]

**Tab.S1 List of clinical tissue samples**

| Sample ID | Specimen Type | Tissue Type |
| --- | --- | --- |
| 2187490-T | LSCC | Laryngeal tissue |
| 2187490-N | Adjacent normal | Para-carcinoma tissue |
| 2190368-T | LSCC | Laryngeal tissue |
| 2190368-N | Adjacent normal | Para-carcinoma tissue |
| 2184991-T | LSCC | Laryngeal tissue |
| 2184991-N | Adjacent normal | Para-carcinoma tissue |
| 2101657-T | LSCC | Laryngeal tissue |
| 2101657-N | Adjacent normal | Para-carcinoma tissue |
| 2208656-T | LSCC | Laryngeal tissue |
| 2208656-N | Adjacent normal | Para-carcinoma tissue |
| 2192015-T | LSCC | Laryngeal tissue |
| 2192015-N | Adjacent normal | Para-carcinoma tissue |
| 2180610-T | LSCC | Laryngeal tissue |
| 2180610-N | Adjacent normal | Para-carcinoma tissue |
| 2192837-T | LSCC | Laryngeal tissue |
| 2192837-N | Adjacent normal | Para-carcinoma tissue |
| 2213184-T | LSCC | Laryngeal tissue |
| 2213184-N | Adjacent normal | Para-carcinoma tissue |
| 2213228-T | LSCC | Laryngeal tissue |
| 2213228-N | Adjacent normal | Para-carcinoma tissue |
| 2217080-T | LSCC | Laryngeal tissue |
| 2217080-N | Adjacent normal | Para-carcinoma tissue |
| 2122024-T | LSCC | Laryngeal tissue |
| 2122024-N | Adjacent normal | Para-carcinoma tissue |
| 1944775-T | LSCC | Laryngeal tissue |
| 1944775-N | Adjacent normal | Para-carcinoma tissue |
| 2238007-T | LSCC | Laryngeal tissue |
| 2238007-N | Adjacent normal | Para-carcinoma tissue |
| 1683414-T | LSCC | Laryngeal tissue |
| 1683414-N | Adjacent normal | Para-carcinoma tissue |
| 2240350-T | LSCC | Laryngeal tissue |
| 2240350-N | Adjacent normal | Para-carcinoma tissue |
| 2214443-T | LSCC | Laryngeal tissue |
| 2214443-N | Adjacent normal | Para-carcinoma tissue |
| 2257522-T | LSCC | Laryngeal tissue |
| 2257522-N | Adjacent normal | Para-carcinoma tissue |
| 2259787-T | LSCC | Laryngeal tissue |
| 2259787-N | Adjacent normal | Para-carcinoma tissue |
| 1714142-T | LSCC | Laryngeal tissue |
| 1714142-N | Adjacent normal | Para-carcinoma tissue |

**Tab. S2 YP-RNS804** **(Hefei Leagor Biotech Corp., Hefei, China)**

| No. | Age | Sex | Organ/Anatomic Site | Pathology diagnosis | Grade | TNM | Stage | Tissue ID. | Type |
| --- | --- | --- | --- | --- | --- | --- | --- | --- | --- |
| 1 | 50 | M | Larynx | Squamous cell carcinoma | 1 | T1N0M0 | I | Rla010227 | Malignant |
| 2 | 50 | M | Larynx | Squamous cell carcinoma | 1 | T1N0M0 | I | Rla010227 | Malignant |
| 3 | 47 | M | Larynx | Squamous cell carcinoma | 1 | T4N0M0 | IVA | Rla020053 | Malignant |
| 4 | 47 | M | Larynx | Squamous cell carcinoma | 1 | T4N0M0 | IVA | Rla020053 | Malignant |
| 5 | 51 | M | Larynx | Squamous cell carcinoma | 1 | T2N0M0 | II | Rla020054 | Malignant |
| 6 | 51 | M | Larynx | Squamous cell carcinoma | 1 | T2N0M0 | II | Rla020054 | Malignant |
| 7 | 55 | M | Larynx | Squamous cell carcinoma | 1 | T2N0M0 | II | 6119C1 | Malignant |
| 8 | 55 | M | Larynx | Squamous cell carcinoma | 1 | T2N0M0 | II | 6119C1 | Malignant |
| 9 | 71 | F | Larynx | Squamous cell carcinoma | 1 | T4N1M0 | IVA | Rla010198 | Malignant |
| 10 | 71 | F | Larynx | Squamous cell carcinoma | 1 | T4N1M0 | IVA | Rla010198 | Malignant |
| 11 | 64 | M | Larynx | Squamous cell carcinoma | 1 | T4N0M0 | IVA | Rla010059 | Malignant |
| 12 | 64 | M | Larynx | Squamous cell carcinoma | 1 | T4N0M0 | IVA | Rla010059 | Malignant |
| 13 | 49 | M | Larynx | Squamous cell carcinoma | 1 | T4N0M0 | IVA | Rla010005 | Malignant |
| 14 | 49 | M | Larynx | Squamous cell carcinoma | 1 | T4N0M0 | IVA | Rla010005 | Malignant |
| 15 | 51 | M | Larynx | Squamous cell carcinoma | 2 | T1N0M0 | I | 175919 | Malignant |
| 16 | 51 | M | Larynx | Squamous cell carcinoma | 1 | T1N0M0 | I | 175919 | Malignant |
| 17 | 50 | M | Larynx | Squamous cell carcinoma | 2 | T4N0M0 | III | Rla010107 | Malignant |
| 18 | 50 | M | Larynx | Squamous cell carcinoma | 1 | T4N0M0 | IVA | Rla010107 | Malignant |
| 19 | 45 | M | Larynx | Squamous cell carcinoma | 1 | T2N0M0 | II | Rla010113 | Malignant |
| 20 | 45 | M | Larynx | Squamous cell carcinoma | 1 | T2N0M0 | II | Rla010113 | Malignant |
| 21 | 45 | M | Larynx | Squamous cell carcinoma | 2 | T4N0M0 | IVA | Rla010163 | Malignant |
| 22 | 45 | M | Larynx | Squamous cell carcinoma | 1 | T4N0M0 | IVA | Rla010163 | Malignant |
| 23 | 71 | M | Larynx | Squamous cell carcinoma | 2 | T3N0M0 | III | Rla020058 | Malignant |
| 24 | 71 | M | Larynx | Squamous cell carcinoma | 2 | T3N0M0 | III | Rla020058 | Malignant |
| 25 | 41 | M | Larynx | Squamous cell carcinoma | 2 | T4N1M0 | IVA | Rln010162 | Malignant |
| 26 | 41 | M | Larynx | Squamous cell carcinoma | 2 | T4N1M0 | IVA | Rln010162 | Malignant |
| 27 | 72 | M | Larynx | Squamous cell carcinoma | 1 | T2N0M0 | II | Rla010202 | Malignant |
| 28 | 72 | M | Larynx | Squamous cell carcinoma | 1 | T2N0M0 | II | Rla010202 | Malignant |
| 29 | 65 | M | Larynx | Squamous cell carcinoma | 2 | T1N0M0 | I | Rla010001 | Malignant |
| 30 | 65 | M | Larynx | Squamous cell carcinoma | 2 | T1N0M0 | I | Rla010001 | Malignant |
| 31 | 70 | F | Larynx | Squamous cell carcinoma | 2 | T3N1M0 | III | 189798B1 | Malignant |
| 32 | 70 | F | Larynx | Squamous cell carcinoma | 2 | T3N1M0 | III | 189798B1 | Malignant |
| 33 | 64 | M | Larynx | Squamous cell carcinoma | 2 | T4N1M0 | IVA | Rla010072 | Malignant |
| 34 | 64 | M | Larynx | Squamous cell carcinoma | 2 | T4N1M0 | IVA | Rla010072 | Malignant |
| 35 | 54 | M | Larynx | Squamous cell carcinoma | 1 | T2N0M0 | II | 63714C3 | Malignant |
| 36 | 54 | M | Larynx | Squamous cell carcinoma | 1 | T2N0M0 | II | 63714C3 | Malignant |
| 37 | 48 | M | Larynx | Squamous cell carcinoma | 2 | T1N0M0 | I | Rla010106 | Malignant |
| 38 | 48 | M | Larynx | Squamous cell carcinoma | 2 | T1N0M0 | I | Rla010106 | Malignant |
| 39 | 54 | M | Larynx | Squamous cell carcinoma (sparse) | 2 | T2N1M0 | III | Rla010006 | Malignant |
| 40 | 54 | M | Larynx | Squamous cell carcinoma | 2 | T2N1M0 | III | Rla010006 | Malignant |
| 41 | 55 | M | Larynx | Squamous cell carcinoma with necrosis | 2 | T2N0M0 | II | 064933C2 | Malignant |
| 42 | 55 | M | Larynx | Squamous cell carcinoma with necrosis | 2 | T2N0M0 | II | 064933C2 | Malignant |
| 43 | 59 | M | Larynx | Squamous cell carcinoma with necrosis | 2 | T4N0M0 | IVA | Rla010109 | Malignant |
| 44 | 59 | M | Larynx | Squamous cell carcinoma | 2 | T4N0M0 | IVA | Rla010109 | Malignant |
| 45 | 54 | F | Larynx | Squamous cell carcinoma | 2 | T2N0M0 | II | 7251C2 | Malignant |
| 46 | 54 | F | Larynx | Squamous cell carcinoma | 2 | T2N0M0 | II | 7251C2 | Malignant |
| 47 | 72 | M | Larynx | Squamous cell carcinoma | 1 | T3N0M0 | III | Rla010173 | Malignant |
| 48 | 72 | M | Larynx | Squamous cell carcinoma | 1 | T3N0M0 | III | Rla010173 | Malignant |
| 49 | 55 | M | Larynx | Squamous cell carcinoma (sparse) | 2 | T2N0M0 | II | 064911C3 | Malignant |
| 50 | 55 | M | Larynx | Squamous cell carcinoma | 2 | T2N0M0 | II | 064911C3 | Malignant |
| 51 | 67 | M | Larynx | Squamous cell carcinoma | 2 | T4N1M0 | IVA | Rla010145 | Malignant |
| 52 | 67 | M | Larynx | Squamous cell carcinoma | 2 | T4N1M0 | IVA | Rla010145 | Malignant |
| 53 | 68 | M | Larynx | Squamous cell carcinoma (gland tissue) | * | T4N0M0 | IVA | Rla010144 | Malignant |
| 54 | 68 | M | Larynx | Squamous cell carcinoma | 2 | T4N0M0 | IVA | Rla010144 | Malignant |
| 55 | 72 | M | Larynx | Squamous cell carcinoma | 2 | T2N1M0 | III | Rla010172 | Malignant |
| 56 | 72 | M | Larynx | Squamous cell carcinoma | 2 | T2N1M0 | III | Rla010172 | Malignant |
| 57 | 64 | M | Larynx | Squamous cell carcinoma | 3 | T2N0M0 | II | Rla010088 | Malignant |
| 58 | 64 | M | Larynx | Squamous cell carcinoma | 3 | T2N0M0 | II | Rla010088 | Malignant |
| 59 | 47 | M | Larynx | Squamous cell carcinoma with necrosis | 3 | T4N1M0 | IVA | Rla020060 | Malignant |
| 60 | 47 | M | Larynx | Squamous cell carcinoma | 3 | T4N1M0 | IVA | Rla020060 | Malignant |
| 61 | 66 | M | Larynx | Adjacent normal larynx tissue | - | - | - | Rla040095 | NAT |
| 62 | 66 | M | Larynx | Adjacent normal larynx tissue | - | - | - | Rla040095 | NAT |
| 63 | 74 | M | Larynx | Adjacent normal larynx tissue | - | - | - | Rla030203 | NAT |
| 64 | 74 | M | Larynx | Adjacent normal larynx tissue | - | - | - | Rla030203 | NAT |
| 65 | 64 | M | Larynx | Adjacent normal larynx tissue | - | - | - | Rla040055 | NAT |
| 66 | 64 | M | Larynx | Adjacent normal larynx tissue | - | - | - | Rla040055 | NAT |
| 67 | 56 | M | Larynx | Adjacent normal larynx tissue with epithelial haperplasia | - | - | - | Rla020784 | NAT |
| 68 | 56 | M | Larynx | Adjacent normal larynx tissue | - | - | - | Rla020784 | NAT |
| 69 | 59 | M | Larynx | Adjacent normal larynx tissue | - | - | - | Rla010002 | NAT |
| 70 | 59 | M | Larynx | Adjacent normal larynx tissue | - | - | - | Rla010002 | NAT |
| 71 | 43 | M | Larynx | Larynx tissue | - | - | - | Rla06N009 | Normal |
| 72 | 43 | M | Larynx | Larynx tissue | - | - | - | Rla06N009 | Normal |
| 73 | 16 | M | Larynx | Larynx tissue | - | - | - | Rla06N007 | Normal |
| 74 | 16 | M | Larynx | Larynx tissue | - | - | - | Rla06N007 | Normal |
| 75 | 45 | M | Larynx | Larynx tissue | - | - | - | Rla06N003 | Normal |
| 76 | 45 | M | Larynx | Larynx tissue | - | - | - | Rla06N003 | Normal |
| 77 | 15 | F | Larynx | Larynx tissue | - | - | - | Rla06N002 | Normal |
| 78 | 15 | F | Larynx | Larynx tissue | - | - | - | Rla06N002 | Normal |
| 79 | 42 | F | Larynx | Larynx tissue | - | - | - | Rla03N006 | Normal |
| 80 | 42 | F | Larynx | Larynx tissue | - | - | - | Rla03N006 | Normal |

**Tab. S3 HN049La01 (Zhongke Guanghua Intelligent Biotech Co., Ltd., Xi'an, China)**

| No. | Age | Gender | Pathological Diagnosis | Grade | TNM | Stage | Tissue Type | Clinical Diagnosis | Lymph Node Metastasis |
| --- | --- | --- | --- | --- | --- | --- | --- | --- | --- |
| A1 | 66 | M | Squamous cell carcinoma (keratinizing) | 1 | T2N0M0 | II | Malignant | Laryngeal cancer | No |
| A2 | 73 | M | Squamous cell carcinoma (keratinizing) | 1 | T2N2bM0 | IVA | Malignant | Laryngeal cancer | Yes |
| A3 | 75 | M | Squamous cell carcinoma (keratinizing) | 1 | T2N0M0 | II | Malignant | Laryngeal cancer | No |
| A4 | 63 | M | Squamous cell carcinoma (keratinizing) | 1 | T3N0M0 | III | Malignant | Laryngeal cancer | No |
| A5 | 66 | M | Squamous cell carcinoma (keratinizing) | 1 | T2N0M0 | II | Malignant | Laryngeal cancer | No |
| A6 | 61 | M | Squamous cell carcinoma (keratinizing) | 1 | T2N1M0 | IIIA | Malignant | Laryngeal cancer | Yes |
| A7 | 64 | M | Squamous cell carcinoma (keratinizing) | 1 | T2N2bM0 | IVA | Malignant | Laryngeal cancer | Yes |
| A8 | 52 | M | Squamous cell carcinoma (keratinizing) | 1 | T1N0M0 | I | Malignant | Laryngeal cancer | No |
| B1 | 74 | M | Squamous cell carcinoma (keratinizing) | 2 | T4N0M0 | IV | Malignant | Laryngeal cancer | No |
| B2 | 67 | M | Squamous cell carcinoma (keratinizing) | 2 | T4aN0M0 | IVA | Malignant | Laryngeal cancer | No |
| B3 | 68 | M | Squamous cell carcinoma (keratinizing) | 2 | T3N0M0 | - | Malignant | Laryngeal cancer | No |
| B4 | 57 | M | Squamous cell carcinoma (keratinizing) | 2 | T2N0M0 | II | Malignant | Laryngeal cancer | No |
| B5 | 60 | M | Squamous cell carcinoma (keratinizing) | 2 | T4aN0M0 | IVA | Malignant | Laryngeal cancer | No |
| B6 | 58 | M | Squamous cell carcinoma (keratinizing) | 2 | T2N0M0 | IB | Malignant | Laryngeal cancer | No |
| B7 | 60 | M | Squamous cell carcinoma (keratinizing) | 2 | T2N0M0 | II | Malignant | Laryngeal cancer | No |
| B8 | 60 | M | Squamous cell carcinoma (keratinizing) | 2 | T3aN1M0 | III | Malignant | Laryngeal cancer | Yes |
| C1 | 69 | M | Squamous cell carcinoma (keratinizing) | 2 | T4aN0M0 | IVA | Malignant | Laryngeal cancer | No |
| C2 | 77 | M | Squamous cell carcinoma (keratinizing) | 2 | T4aN2cM0 | IVA | Malignant | Laryngeal cancer | Yes |
| C3 | 67 | M | Squamous cell carcinoma (non-keratinizing) | 2 | T2N1M0 | IIIA | Malignant | Laryngeal cancer | Yes |
| C4 | 69 | M | Squamous cell carcinoma (keratinizing) | 2 | T2N0M0 | II | Malignant | Laryngeal cancer | No |
| C5 | 51 | M | Squamous cell carcinoma (keratinizing) | 2 | T3N2cM0 | IVA | Malignant | Laryngeal cancer | Yes |
| C6 | 75 | M | Squamous cell carcinoma (non-keratinizing) | 2 | T3N2bM0 | IVA | Malignant | Laryngeal cancer | Yes |
| C7 | 55 | M | Squamous cell carcinoma (keratinizing) | 2 | T2N0M0 | II | Malignant | Laryngeal cancer | No |
| C8 | 64 | M | Squamous cell carcinoma (keratinizing) | 2 | T3N0M0 | III | Malignant | Laryngeal cancer | No |
| D1 | 65 | M | Squamous cell carcinoma (keratinizing) | 2 | T4aN0M0 | IVA | Malignant | Laryngeal cancer | No |
| D2 | 64 | M | Squamous cell carcinoma (non-keratinizing) | 2 | T3N1M0 | III | Malignant | Laryngeal cancer | Yes |
| D3 | 53 | M | Squamous cell carcinoma (keratinizing) | 2 | T2N2bM0 | IVA | Malignant | Laryngeal cancer | Yes |
| D4 | 67 | M | Squamous cell carcinoma (non-keratinizing) | 2 | T2N2cM1 | IVC | Malignant | Laryngeal cancer | Yes |
| D5 | 67 | M | Squamous cell carcinoma (non-keratinizing) | 2 | T3N2cM0 | IVA | Malignant | Laryngeal cancer | Yes |
| D6 | 66 | M | Squamous cell carcinoma (non-keratinizing) | 2 | T3N1M0 | III | Malignant | Laryngeal cancer | Yes |
| D7 | 53 | M | Squamous cell carcinoma (keratinizing) | 2 | T3N0M0 | III | Malignant | Laryngeal cancer | No |
| D8 | 45 | M | Squamous cell carcinoma (non-keratinizing) | 3 | T2N0M0 | II | Malignant | Laryngeal cancer | No |
| E1 | 62 | M | Squamous cell carcinoma (keratinizing) | 2-3 | T1N0M0 | I | Malignant | Laryngeal cancer | No |
| E2 | 60 | M | Squamous cell carcinoma (non-keratinizing) | 3 | T2N0M0 | II | Malignant | Laryngeal cancer | No |
| E3 | 72 | M | Squamous cell carcinoma (non-keratinizing) | 3 | T2N2aM0 | IVA | Malignant | Laryngeal cancer | Yes |
| E4 | 60 | M | Squamous cell carcinoma (keratinizing) | 3 | T3N0M0 | III | Malignant | Laryngeal cancer | No |
| E5 | 51 | M | Squamous cell carcinoma (non-keratinizing) | 3 | T1N0M0 | I | Malignant | Laryngeal cancer | No |
| E6 | 63 | M | Squamous cell carcinoma (non-keratinizing) | 3 | T3N0M0 | III | Malignant | Laryngeal cancer | No |
| E7 | 47 | M | Squamous cell carcinoma (non-keratinizing) | 3 | T2N0M0 | - | Malignant | Laryngeal cancer | No |
| E8 | 76 | M | Squamous cell carcinoma (non-keratinizing) | 3 | T1N0M0 | I | Malignant | Laryngeal cancer | No |
| F1 | 43 | M | Squamous cell carcinoma (non-keratinizing) | 3 | T2N1M0 | III | Malignant | Laryngeal cancer | Yes |
| F2 | 56 | M | Squamous cell carcinoma (keratinizing) | 2-3 | T3N0M0 | III | Malignant | Laryngeal cancer | No |
| F3 | 59 | M | Squamous cell carcinoma (non-keratinizing) | 3 | T1N0M0 | I | Malignant | Laryngeal cancer | No |
| F4 | 60 | M | Moderate dysplasia of laryngeal squamous epithelium | - | - | - | Adjacent normal | - | - |
| F5 | 77 | M | Severe dysplasia of laryngeal squamous epithelium (carcinoma in situ) | - | - | - | Adjacent normal | - | - |
| F6 | 40 | M | Laryngeal tissue | - | - | - | Normal | - | - |
| F7 | 33 | M | Laryngeal tissue | - | - | - | Normal | - | - |
| F8 | 40 | F | Laryngeal tissue | - | - | - | Normal | - | - |
| F9 | 37 | M | Laryngeal tissue | - | - | - | Normal | - | - |
