# Supplementary material for: The MYBL2-GTSE1 axis promotes laryngeal squamous cell carcinoma progression by regulating PI3K/AKT-dependent glycolytic reprogramming
Source: Cancer Biol Ther. 2026 Mar 22;27(1):2648193. doi: 10.1080/15384047.2026.2648193 (PMC13011630; doi:10.1080/15384047.2026.2648193)
Supplement: Supplementary File 1.docx [file KCBT_A_2648193_SM0569.docx]

**Fig.S1** **Lentiviral transduction efficiency of MYBL2 knockdown constructs.**
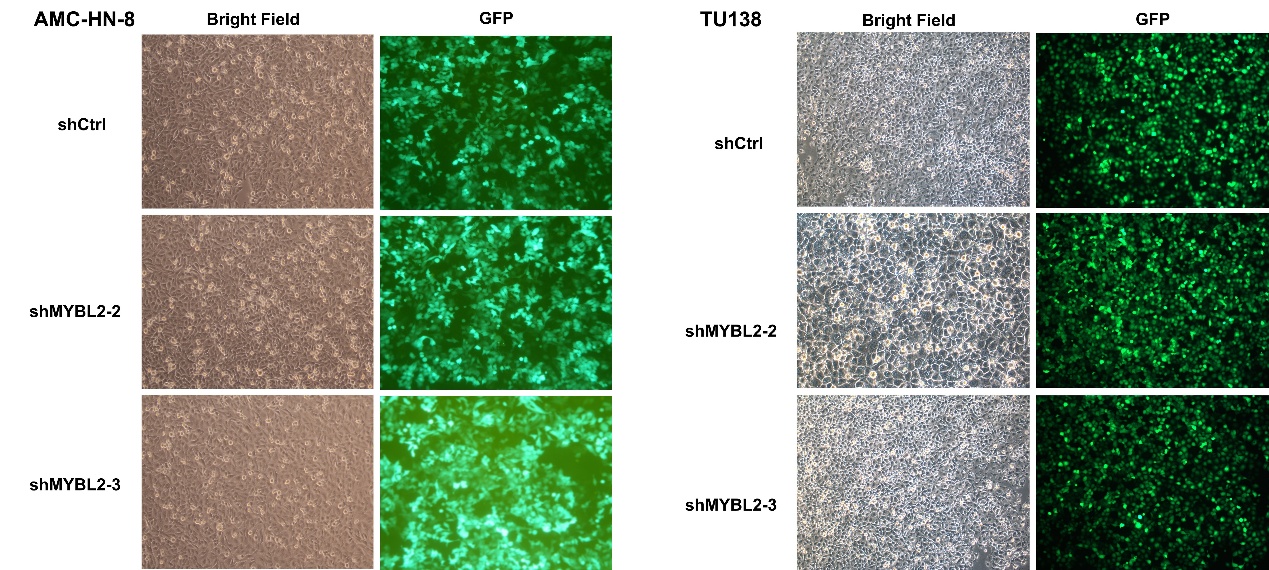


**Representative bright-field and GFP fluorescence images of AMC-HN-8 and TU138 cells transduced with shCtrl, shMYBL2-2 and shMYBL2-3. High GFP positivity confirms successful viral transduction.**

**Fig.S2 Transduction efficiency for combined MYBL2 overexpression and GTSE1 knockdown.**


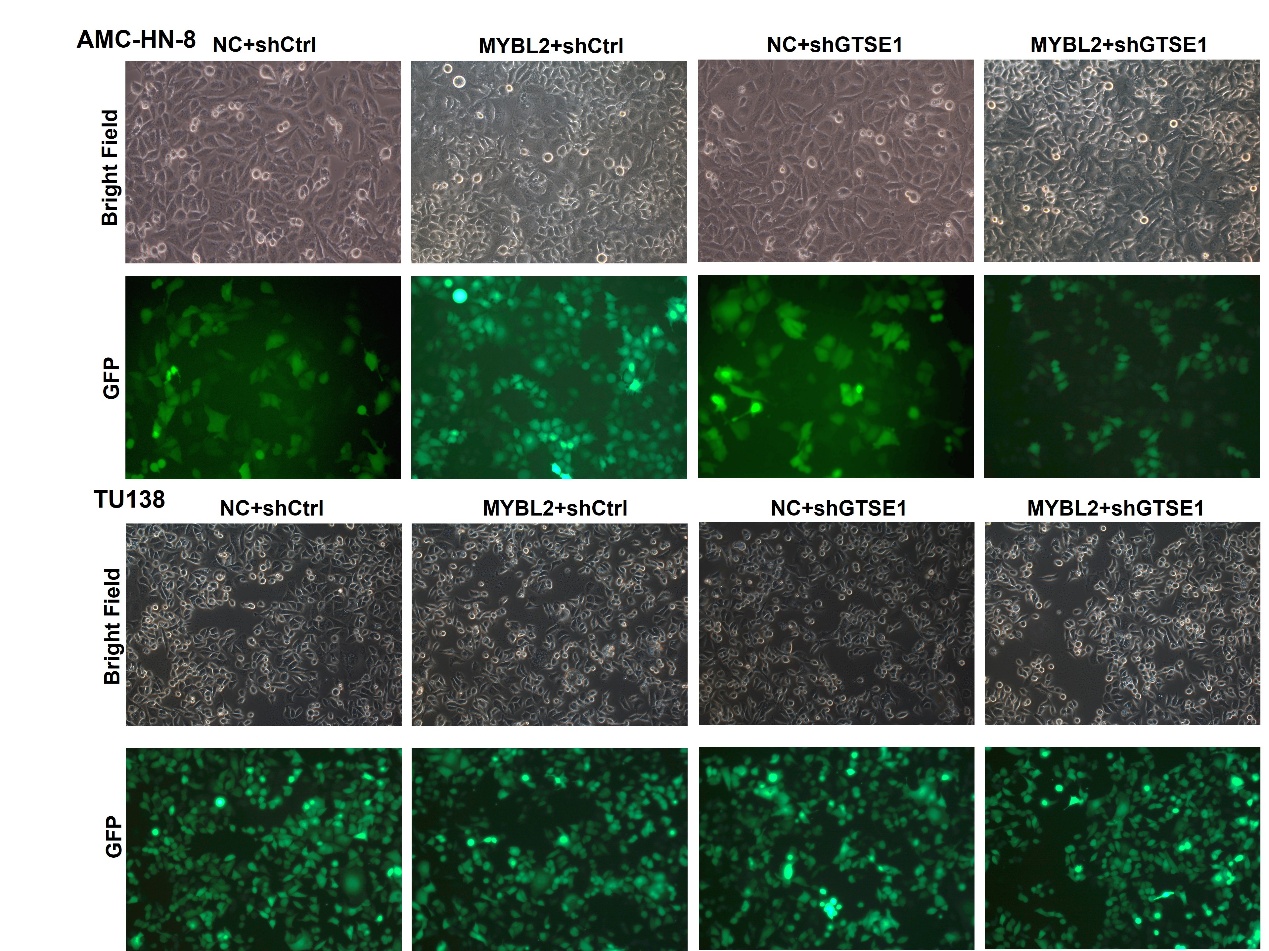


**Representative images of AMC-HN-8 and TU138 cells co-transduced with the indicated combinations: NC+shCtrl, MYBL2+shCtrl, NC+shGTSE1, and MYBL2+shGTSE1. GFP expression confirms successful dual transduction.**

**Fig. S3 Validation of MYBL2-mediated glycolytic regulation by AKT inhibition.**


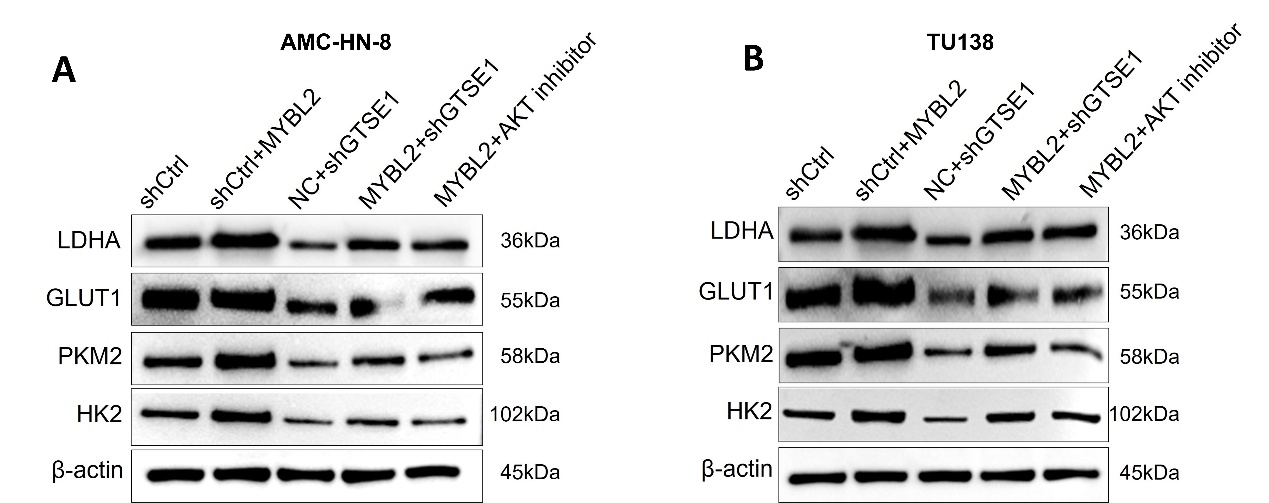
**A–B. Western blot analysis of glycolytic proteins (LDHA, GLUT1, PKM2, HK2) in AMC-HN-8 and TU138 cells following MYBL2 overexpression alone or combined with GTSE1 knockdown/AKT inhibitor (MK-2206, 5μM, 24h) treatment. Notably, MK-2206 treatment effectively reversed the upregulation of glycolytic proteins induced by MYBL2, yielding results consistent with those of PI3K inhibition.**

**Fig. S4. Validation of apoptosis detection with positive control in AMC-HN-8 cells.**
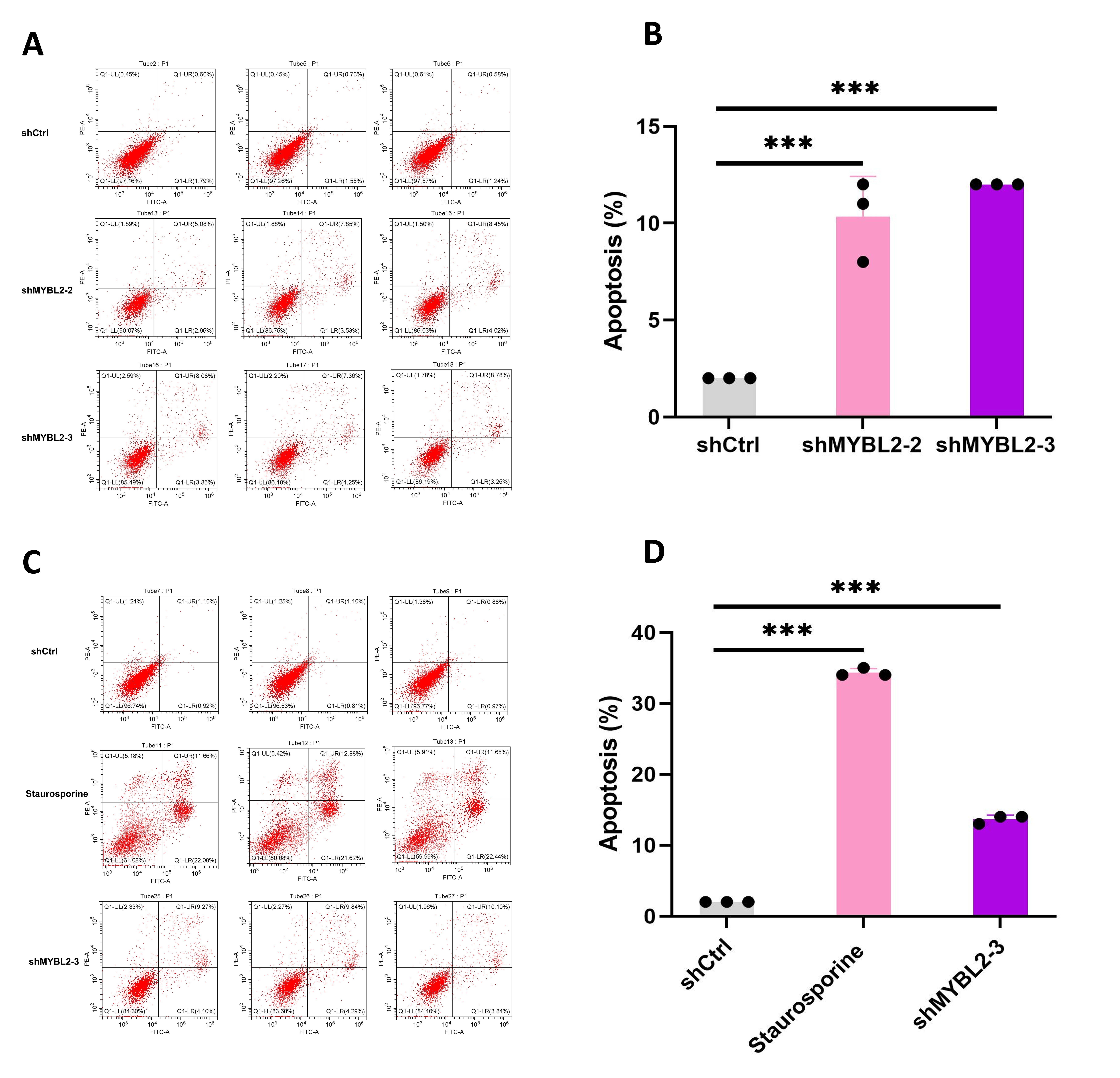


**A–B. Annexin V-FITC/PI staining and flow cytometry analysis of AMC-HN-8 cells transduced with shCtrl, shMYBL2-2, or shMYBL2-3, showing representative plots (A) and quantification of apoptosis rates (B). C–D. Positive control validation with Staurosporine (STS, 1 μM, 24 h) showing representative plots (C) and quantification (D). Data are presented as mean ± SD, n = 3, *** *P* < 0.001.**

**Fig. S5 MYBL2 Knockdown in TU686 Cells Demonstrates Suppression of Proliferation and Migration.**


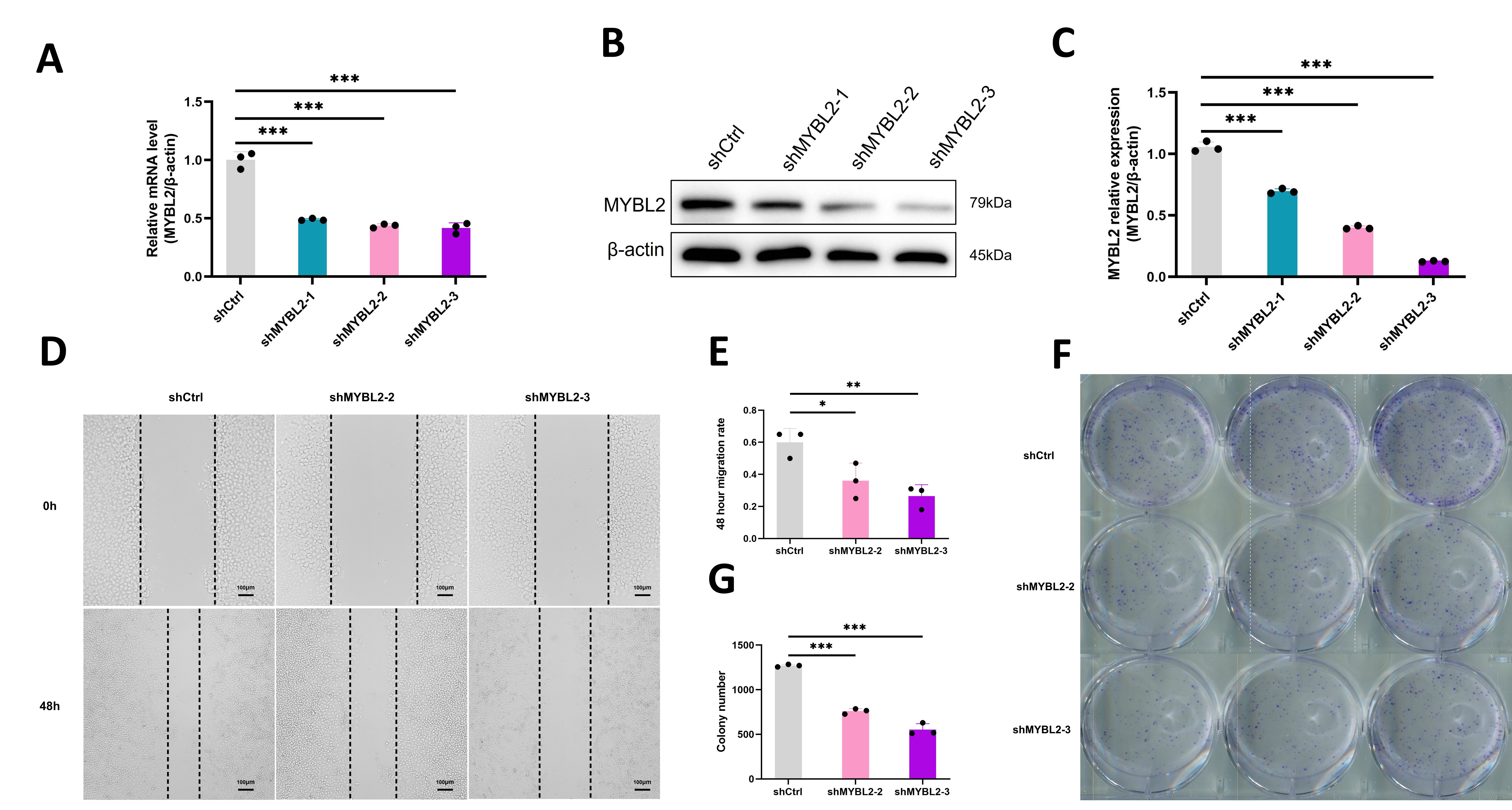


**A–C. Knockdown efficiency confirmation in TU686 cells by qRT-PCR (A) and Western blot (B) after lentiviral transduction of shMYBL2-1, shMYBL2-2, and shMYBL2-3. Quantification of MYBL2 protein levels normalized to β-actin (C). D–E. Wound healing assays showing the migration capability of TU686 cells transduced with shMYBL2-2 and shMYBL2-3 (scale bar, 100 μm). F–G. Colony formation assays demonstrating the clonogenic potential of TU686 cells transduced with shMYBL2-2 and shMYBL2-3. Data are presented as mean ± SEM, n = 3. * *P* < 0.05, ** *P* < 0.01, *** *P* < 0.001.**

**Fig. S6 Functional validation of shGTSE1 by rescue experiments in AMC-HN-8 cells.**
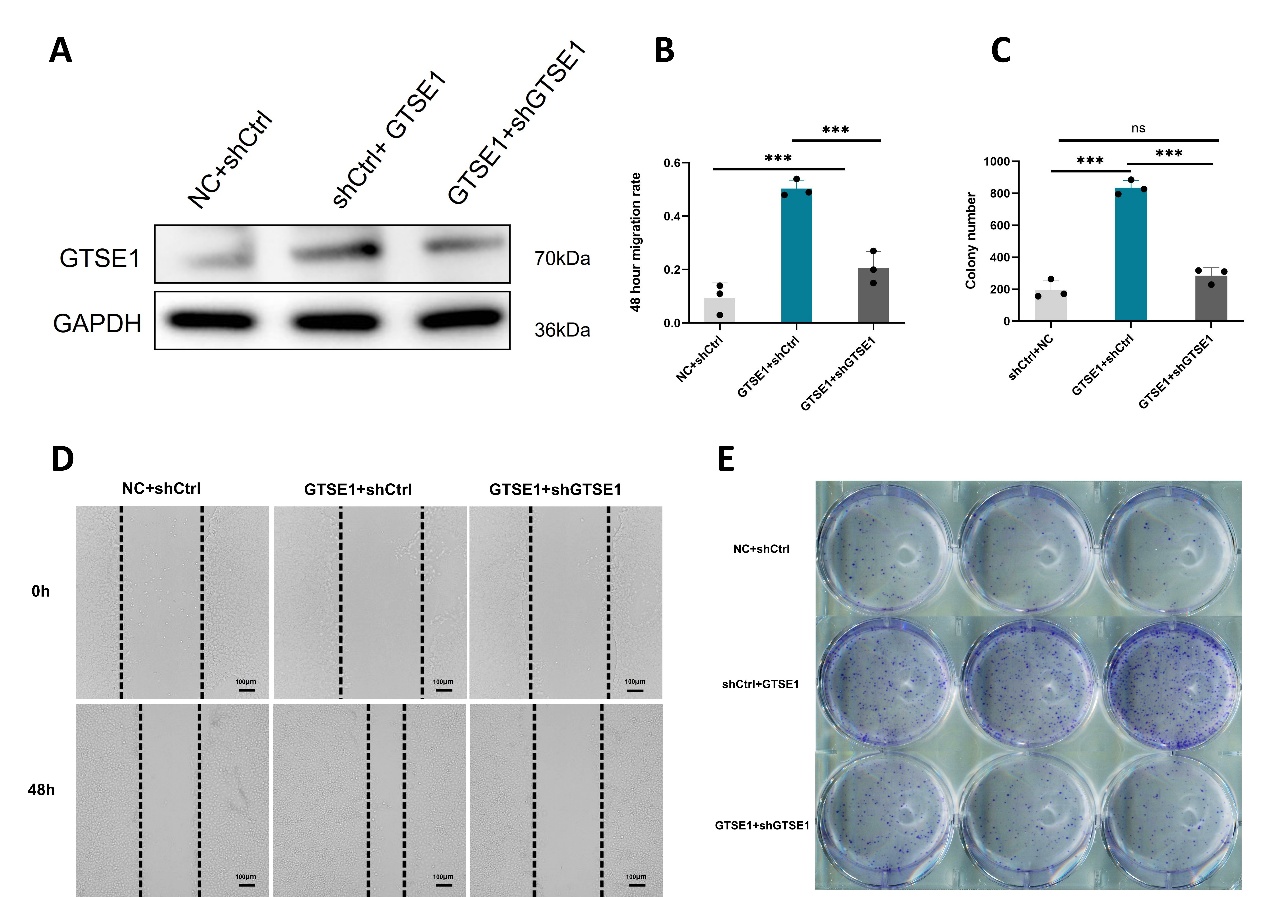
**A. Western blot analysis of GTSE1 protein levels in AMC-HN-8 cells transduced with control (NC+shCtrl), GTSE1-overexpressing (shCtrl+GTSE1), and rescue (GTSE1+shGTSE1)*.* B–C. Quantification of migration rate at 48 h (B) and colony numbers (C). D–E. Representative images of wound healing (D, scale bar: 100 μm) and colony formation assays of AMC-HN-8 cells (E). Data are mean ± SEM, n = 3. ns, not significant; *** *P* < 0.001.**

**Tab.S1 primary antibody information**

| **Antibody Name** | **Destination Strip Size**  **kDa** | **Dilution multiple** | **Source of primary antibody** | **Company** | **Cat. No** |
| --- | --- | --- | --- | --- | --- |
| MYBL2 | 79/90 | 1:500 | Rabbit | Proteintech | 18896-1-AP |
| GTSE1 | 70 | 1:1000 | Rabbit | ABclonal | A13902 |
| PKM2 | 58 | 1:5000 | Rabbit | Proteintech | 15822-1-AP |
| HK2 | 102 | 1:3000 | Mouse | Proteintech | 66974-1-Ig |
| GLUT1 | 54 | 1:1000 | Rabbit | Proteintech | 21829-1-AP |
| LDHA | 36 | 1:3000 | Rabbit | Abcam | ab52488 |
| PI3K | 110 | 1:1000 | Rabbit | CST | 4249S |
| p-PI3K | 60,85 | 1:1000 | Rabbit | CST | 17366S |
| AKT | 56 | 1:3000 | Rabbit | Proteintech | 10176-2-AP |
| p-AKT | 60 | 1:5000 | Mouse | Proteintech | 66444-1-Ig |
| GAPDH | 36 | 1:30000 | Mouse | Proteintech | 60004-1-lg |
| β-Actin | 42/45 | 1:2000 | Rabbit | Proteintech | 20536-1-AP |

**Tab.S2** **secondary antibody information**

| **Antibody Name** | **Dilution multiple** | **Company** | **Cat. No** |
| --- | --- | --- | --- |
| Goat Anti-Rabbit | 1:3000 | Beyotime | A0208 |
| Goat Anti-Mouse | 1:3000 | Beyotime | A0216 |

**Tab.S3 Isotype Control**

| **Antibody Name** | **Application** | **Company** | **Cat.No** |
| --- | --- | --- | --- |
| Rabbit IgG Control | matched to MYBL2 antibody | Abclonal | AC005 |

**Tab.S4** **Primer information**

| **GENE** | **Forward primer (5’-3’)** | **Reverse primer (5’-3’)** |
| --- | --- | --- |
| MYBL2 | CTTGAGCGAGTCCAAAGACTG | AGTTGGTCAGAAGACTTCCCT |
| GTSE1 | CCACCGGGATGTTCTCCCT | TTCAGCCCCAACTTGTTTGGA |
| GAPDH | TGACTTCAACAGCGACACCCA | CACCCTGTTGCTGTAGCCAAA |
| β-actin | CAAAGTTCACAATGTGGCCGAGGA | GGGACTTCCTGTAACAACGCATCT |
|  |  |  |

**Tab.S5** **tissue microarrays (TMAs) Information**

| Company：Hefei Daxiang Biotechnology Group Co., Ltd. | Type:YP-RNS804（60 tumor+20 adjacent normal） |
| --- | --- |
| Company：Zhongke Guanghua (Xi'an) Intelligent Biotechnology Co., Ltd. | Type:HN049La01（43tumor +6 adjacent normal） |
| Antibody information：MYBL2 | Dilution multiple: 1：100 |
| Company：abcam | Cat.No：ab191064 |

**Remarks:** Number of cancerous desquamation: 4 cases (YP-RNS804: 2 cases; HN049La01: 2 cases); number of adjacent normal desquamation: 1 case (YP-RNS804); number of incomplete pathological data: 3 cases.

**Tab.S6 Main cell lines**

| **Equipment Name** | **Cat. No** | **Company** |
| --- | --- | --- |
| AMC-HN-8 | TCHu262 | Cell Bank of the Chinese Academy of Science |
| TU138 | CVCL 4910 | BTCC-1192 |
| HPLMEC | RPC-050 | RUNTOGGN |
| TU686 | ORC0873 | Chemical Book |
| TU212 | SNL-497 | SUNCELL |

**Tab.S7** **Reagents and Drug Information**

| **Trial** | **Reagents** |  | **Cat. No** | **Company** |
| --- | --- | --- | --- | --- |
| Screening reagents | Puromycin |  | ST551 | Beyotime |
| RNA Analysis | TRIzol |  | R0016 | Beyotime |
|  | RevertAid First Strand cDNA Synthesis Kit |  | K1621 | Thermo Fisher Scientific |
|  | Bestar SYBR Green Mastermix Kit |  | DBI-2073 | DBI Bioscience |
| Protein Analysis | RIPA lysis buffer |  | P0013B | Beyotime |
|  | Phosphatase Inhibitor |  | P1045 | Beyotime |
|  | PVDF membrane |  | IPVH00010 | Merck |
|  | Tween-20 |  | ST828 | Beyotime |
|  | DAB substrate kit |  | ab64238 | Abcam |
|  | ECL Colorimetric Substrate Kit |  | P0018S | Beyotime |
|  | High Purity BioReagent |  | ST2067-20g | Beyotime |
|  | eosin |  | C0109 | Beyotime |
| Function trial | PI3K inhibitor LY294002 |  | S1737 | Beyotime |
|  | AKT inhibitor  MK-2206 |  | S1078 | Selleck |
|  | Mitomycin C |  | 50-07-7 | MedChemExpress |
|  | Staurosporine |  | HY-15141 | MedChemExpress |
|  | Matrigel |  | C0383 | Beyotime |
|  | Matrigel Matrix |  | BD 356234 | Corning |
|  | Annexin V-APC/PI Apoptosis Kit |  | E-CK-A217 | Elabscience |
|  | Glycolytic Stress Test Kit |  | BB-48311 | BestBio |
|  | Mitochondrial Stress Test Kit |  | BB-48211 | BestBio |
|  | 4% paraformaldehyde |  | B1057 | Applygen |
|  | Crystal Violet |  | G1062 | Solarbio |
|  | Cell Counting Kit-8 |  | C0038 | Beyotime |
| molecular clone | pGL3-basic reporter vector |  | E1751 | Promega |
|  | Internal Reference Reporter Vector pRL-TK |  | E2241 | Promega |
|  | Dual-Luciferase Reporter Assay Kit |  | RG027 | Beyotime |
|  | Protein A-Agarose Beads |  | 1614813 | BIO-RAD |
| General Reagents | DMSO |  | Y026158 | Beyotime |
|  | DMEM |  | L1006-500 | BDBIO |
|  | 1640 |  | L1039-500 | BDBIO |
|  | Phosphate Buffer (PBS) |  | BL302A | Biosharp |
|  | Trypsin |  | 25200056 | Thermo Fisher Scientific |
|  | Fetal Bovine Serum |  | VS500T | Ausbian |
|  | Penicillin-Streptomycin-Gentamicin Solution |  | P1410 | Solarbio |

**Tab.S8 Main Equipment**

| **Equipment Name** | **Cat. No** | **Company** |
| --- | --- | --- |
| 96-well plate | 3596 | Corning |
| 6-well plate | 3516 | Corning |
| 12-well plate | 3513 | Corning |
| Cellometer Mini Cell Counter | Mini-006-0635 | Nexcelom |
| Microplate Reader | M2009PR | Tecan infinite |
| Florescence Microscope | IX73 | Olympus |
| Inverted Microscope | CKX31 | Olympus |
| Biohazard Safety Equipment | HFsafe-1500Lc | Heal-Force |
| Centrifugal Machine | Fresco 21 | Thermo Fisher Scientific |
| CO2 Incubator | 371 | Thermo Fisher Scientific |
| Celigo Image Cytometer | BFFL-5C | Nexcelom |
