# Supplementary material for: The MYBL2-GTSE1 axis promotes laryngeal squamous cell carcinoma progression by regulating PI3K/AKT-dependent glycolytic reprogramming
Source: Cancer Biol Ther. 2026 Mar 22;27(1):2648193. doi: 10.1080/15384047.2026.2648193 (PMC13011630; doi:10.1080/15384047.2026.2648193)
Supplement: Supplementary Table S2.docx [file KCBT_A_2648193_SM3072.docx]

**Supplementary Table S2. Univariate Cox regression analysis of 30 candidate genes associated with LSCC prognosis.**

| **Symbol** | **GeneType** | **log_2_FC** | ***P* val.** | **log_2_FC.exp** | **Adj. *P* val. exp** | **HR (95% CI)** | ***P* val.**  **(os)** |
| --- | --- | --- | --- | --- | --- | --- | --- |
| MYBL2 | protein_coding | 1.541693 | 0.00063 | 2.068119 | 3.21E-05 | 4.99(2.21-11.27) | 2.20E-05 |
| CDKN3 | protein_coding | 1.094054 | 0.002729 | 1.461767 | 5.57E-05 | 3.24(1.51-6.95) | 0.00147 |
| HOXC13 | protein_coding | 3.282672 | 0.000139 | 5.742493 | 3.39E-05 | 2.93(1.46-5.87) | 0.00155 |
| ANO1 | protein_coding | 1.082569 | 0.000901 | 3.295281 | 0.001436 | 2.63(1.3-5.31) | 0.00531 |
| SLC7A5 | protein_coding | 2.118593 | 1.46E-06 | 1.740531 | 0.000608 | 2.79(1.31-5.92) | 0.00561 |
| ADAM12 | protein_coding | 2.200669 | 2.11E-07 | 4.774141 | 3.39E-05 | 2.62(1.25-5.47) | 0.00779 |
| MMP1 | protein_coding | 3.662167 | 4.68E-08 | 4.697435 | 3.87E-05 | 2.5(1.24-5.04) | 0.00858 |
| SERPINE1 | protein_coding | 1.903873 | 6.40E-05 | 3.035694 | 0.000241 | 2.42(1.2-4.92) | 0.0114 |
| CCL20 | protein_coding | 1.38321 | 0.019524 | 3.066023 | 0.00989 | 2.33(1.16-4.68) | 0.0144 |
| FOXM1 | protein_coding | 1.511746 | 0.000346 | 2.100016 | 9.24E-05 | 2.34(1.16-4.74) | 0.0152 |
| RFC4 | protein_coding | 1.081838 | 3.12E-06 | 2.124934 | 3.21E-05 | 2.28(1.15-4.52) | 0.0153 |
| ARSI | protein_coding | 2.881476 | 3.95E-09 | 3.35212 | 0.001149 | 2.37(1.15-4.89) | 0.0167 |
| FEN1 | protein_coding | 1.023817 | 4.42E-06 | 1.440542 | 3.87E-05 | 2.24(1.12-4.46) | 0.019 |
| STC2 | protein_coding | 1.162067 | 0.009459 | 4.039603 | 3.21E-05 | 2.18(1.1-4.35) | 0.023 |
| CDT1 | protein_coding | 1.299773 | 0.000111 | 1.53662 | 8.41E-05 | 2.16(1.08-4.3) | 0.0255 |
| RGS20 | protein_coding | 2.487841 | 1.75E-06 | 1.975445 | 0.001149 | 2.19(1.08-4.45) | 0.0269 |
| TK1 | protein_coding | 1.333851 | 0.000426 | 1.921516 | 3.21E-05 | 2.23(1.07-4.66) | 0.0282 |
| MMP13 | protein_coding | 4.378269 | 1.97E-09 | 6.208066 | 0.000348 | 2.1(1.06-4.16) | 0.0287 |
| CENPN | protein_coding | 1.037004 | 9.18E-05 | 1.534877 | 0.000157 | 2.14(1.05-4.34) | 0.0318 |
| NUDT11 | protein_coding | 1.420912 | 4.91E-05 | 2.883318 | 0.001527 | 2.18(1.05-4.57) | 0.0332 |
| CDCA3 | protein_coding | 1.177261 | 0.001921 | 1.819835 | 4.12E-05 | 2.18(1.04-4.56) | 0.0351 |
| COL5A2 | protein_coding | 1.301266 | 0.000319 | 3.3123 | 5.57E-05 | 2.07(1.03-4.18) | 0.037 |
| CTHRC1 | protein_coding | 1.891328 | 6.08E-06 | 4.357183 | 3.21E-05 | 2.03(1.02-4.05) | 0.0391 |
| COL1A1 | protein_coding | 1.815425 | 5.37E-05 | 2.837576 | 8.99E-05 | 2.06(1.02-4.15) | 0.0396 |
| SHCBP1 | protein_coding | 1.443389 | 0.00011 | 1.603346 | 6.63E-05 | 2.04(1.02-4.08) | 0.0401 |
| NETO2 | protein_coding | 1.777042 | 1.92E-06 | 2.609159 | 3.69E-05 | 2.03(1.02-4.05) | 0.0403 |
| UBE2C | protein_coding | 1.264433 | 0.005428 | 1.760484 | 5.29E-05 | 2.08(1.01-4.26) | 0.0428 |
| DHRS2 | protein_coding | 2.385789 | 8.85E-07 | 5.469495 | 0.000581 | 2.05(1-4.18) | 0.0443 |
| LAMP3 | protein_coding | 1.873668 | 0.000164 | 2.281935 | 0.000161 | 2(1-3.99) | 0.0452 |

**Abbreviations: log_2_FC**, log_2_ fold change; **Adj. P val**, adjusted *P* value; **HR**, hazard ratio; **CI**, confidence interval; O**S**, overall survival.
**Note:** The first set of log_2_FC and *P* val. represents the initial screening data, while log_2_FC.exp and Adj. *P*.val.exp represents the validation dataset. Genes are ranked by HR in descending order.
